# Traditional and Innovative Anti-seizure Medications Targeting Key Physiopathological Mechanisms: Focus on Neurodevelopment and Neurodegeneration

**DOI:** 10.2174/1570159X21666230504160948

**Published:** 2023-06-15

**Authors:** Miriam Sciaccaluga, Gabriele Ruffolo, Eleonora Palma, Cinzia Costa

**Affiliations:** 1 Section of Neurology, S.M. della Misericordia Hospital, Department of Medicine and Surgery, University of Perugia, Piazzale Gambuli 1, Perugia, 06129, Italy;; 2 Department of Physiology and Pharmacology, Istituto Pasteur—Fondazione Cenci Bolognetti, University of Rome, Sapienza, Rome, 00185, Italy;; 3 IRCCS San Raffaele Roma, Rome, 00166, Italy

**Keywords:** Epilepsy, ASMs, neuroprotection, neurodevelopment, neurodegeneration, brain ischemia

## Abstract

Despite the wide range of compounds currently available to treat epilepsy, there is still no drug that directly tackles the physiopathological mechanisms underlying its development. Indeed, anti-seizure medications attempt to prevent seizures but are inefficacious in counteracting or rescuing the physiopathological phenomena that underlie their onset and recurrence, and hence do not cure epilepsy. Classically, the altered excitation/inhibition balance is postulated as the mechanism underlying epileptogenesis and seizure generation. This oversimplification, however, does not account for deficits in homeostatic plasticity resulting from either insufficient or excessive compensatory mechanisms in response to a change in network activity. In this respect, both neurodevelopmental epilepsies and those associated with neurodegeneration may share common underlying mechanisms that still need to be fully elucidated. The understanding of these molecular mechanisms shed light on the identification of new classes of drugs able not only to suppress seizures, but also to present potential antiepileptogenic effects or “disease-modifying” properties.

## 1 INTRODUCTION

Epilepsy is the most common chronic neurological disease, affecting around 50 million people worldwide of any age, although it is more frequent in children and the elderly [1].

On the one hand, the developing brain of an infant is in a state in which any kind of insult might have watershed consequences on brain function and histological development. Certainly, many neurodevelopmental conditions are characterized by a strong association with seizures and epilepsy. Furthermore, these patients are frequently burdened also by comorbidities that may have even more incapacitating outcomes than epilepsy itself. In fact, it was reported that attention problems, thought disturbances and impaired social behavior were significantly more frequent in epileptic children in comparison with control groups [2]. Additionally, recent studies also support a close correlation between epilepsy and the physiological mechanisms underlying nervous system development, correlating with different degrees of cognitive and neurobehavioral deficits affecting patients with epilepsy during childhood [3-5].

On the other hand, several neurological conditions that are more frequent in older people, including Alzheimer's disease (AD), brain tumors, stroke, brain trauma, cerebrovascular pathologies or central nervous system (CNS) infections, are also closely intertwined with seizures. Indeed, neurological comorbidities are relatively common in epilepsy, affecting about 50% of patients. In this respect, epilepsy and some neurological comorbidities, such as stroke and dementia, maintain a complex bidirectional relationship, with comorbidities exacerbating epilepsy and *vice versa* [3, 6]. In this scenario, it is possible to hypothesize that epilepsy and comorbid neurologic conditions, both in the case of neurodevelopmental epilepsies and those associated with neurodegeneration, may share common underlying mechanisms that still need to be fully elucidated [7, 8].

It is widely accepted, though it is an oversimplified interpretation, that epilepsy pathophysiology derives from an unbalance between excitatory and inhibitory neurotransmission due to a selective loss of GABA-ergic neurons that causes a reorganization of neural circuits leading to the over-synchronization of neural networks [9]. Although the precise mechanisms and cascade of events driving seizures still need to be fully elucidated, several specific pathophysiologic abnormalities have been identified that promote this disease at different levels. Among these, decreased GABA synthesis or abnormal GABA receptor (GABAR) subunit composition, over- activation of NMDA receptors (NMDARs), decreased levels of GluR2-bearing AMPA receptors (AMPARs) and defects in AMPARs trafficking and turnover, ion channel mutations, contribute to structural and functional circuit impairment [10]. A number of different genetic mutations involving more than 400 genes [11] have been associated with epilepsy. Nevertheless, a substantial number of these mutations also have broader effects on neurological function and cognitive ability. In addition to the well-known mutations in ion channels, GABARs and NMDARs, several genes involved in epilepsy have been identified as risk factors for neurodevelopmental and neurodegenerative disorders [12], including genes controlling cellular metabolism (such as SLC2A1, EPM2A, EPM2B, TPP1, ALDH7A1) and signaling pathways (such as MTOR, AKT3, PIK3CA, TSC1, TSC2, PTEN, DEPDC5, NPRL2, NPRL3) [13]. The identification of the genetic aetiology in individual patients with neurodevelopmental or neurodegenerative disorders can be a useful tool for personalized therapies that can be not only limited to conventional anti-seizure medications (ASMs) but can include compounds that modulate specific metabolic defects (*e.g*., the ketogenic diet for GLUT1 deficiency). In addition, novel therapeutic approaches, including antibody-enzyme fusions, antisense oligonucleotides, and gene therapy are currently in development or in trial to treat rare genetic epilepsies associated with cognitive deterioration.

Until now, the pharmacological agents used to treat seizures have been devised with quite a simple but efficacious objective: to reduce the excessive excitatory stimuli by quenching the depolarizing neurotransmission or boosting its inhibitory potential. It is not surprising that the first two drugs used in history for the treatment of seizures, bromide salts and phenobarbital [14], were both capable of enhancing the function of GABAergic inhibition, thus re-establishing the equilibrium of the “excitation/inhibition” (E/I) scale. Of course, at present days, there is a much wider choice of medications and respective mechanisms of action, with more than 20 ASMs clinically available [15]. ASMs do not cure epilepsy but rather attempt to prevent seizures by acting on multiple neuronal targets, both pre- and post-synaptic [16]. The final common goal of ASMs is to counteract hyperexcitability by either enhancing neuronal inhibition or reducing excitatory neurotransmission [15, 17, 18]. Although most ASMs exhibit multiple mechanisms of action, some of which are not yet fully understood, three basic mechanisms have been recognized at the cellular level: 1) modulation of voltage-gated ion (Na^+^, K^+^, Ca^2+^) channels to counteract sustained repetitive firing in individual neurons; 2) enhancement of GABA-mediated neurotransmission and synaptic pathways; and, 3) modulation of glutamate-mediated neurotransmission.

Moreover, the effects of several ASMs on molecular cascades contributing to neurodegeneration suggest that these compounds may have broader functions than simply suppressing neuronal excitability. Neurodegeneration is an important neurobiological abnormality in the epileptic brain, and it is also a common feature between epilepsy and several comorbidities of epilepsy. Indeed, neuron loss implies a series of neurophysiological alterations, such as axonal and dendritic plasticity dysregulation, molecular reorganization of cell membranes, neurogenesis and gliosis [19]. Since epilepsy and progressive neurodegeneration seem to be closely intertwined, neuroprotection is likely to play a crucial role in the management of the disease and its comorbidities. Indeed, the preservation of neuronal physiology against an ongoing neurodegenerative insult also constitutes a possible treatment strategy to prevent or slow down the progression of several neurodegenerative disorders, such as AD, Parkinson’s disease (PD), and Huntington’s disease (HD), and also multiple sclerosis (MS) [19-22]. In this respect, ASMs have been demonstrated as neuroprotective in experimental models of cerebral ischemia, PD, AD, and brain tumors.

In this review, we will provide an overview of the most relevant data on the neuroprotective effects exerted by ASMs in experimental models of neuronal damage and epilepsies associated with neurodevelopmental impairment, focusing on mechanisms other than the anticonvulsant one. The characterization of these molecular mechanisms may constitute a useful tool to identify new classes of drugs able not only to suppress seizures but also to protect neurons against injuries specifically and to develop potential antiepileptic “disease-modifying” therapies.

## 2 NEURODEVELOPMENT

It is now widely recognized that epilepsy and nervous system development have a tight relationship. Recent studies and data supporting this association come from different perspectives.

First of all, the clinical epileptologists have thoroughly described how different degrees of cognitive impairment may affect patients with epilepsy [3] and how seizures during childhood are often associated with a plethora of neurobehavioral consequences [4], including attention-deficit/hyperactivity disorder (ADHD) [5], autism spectrum disorder (ASD), and serious cognitive impairment in terms of low QI measured through standardized tests (such as the Wechsler Intelligence Scale for children).

On the other hand, evidence from basic science provided a solid ground for the aforementioned association to stand on, most of which is based on the E/I imbalance hypothesis. In several neurodevelopmental pathologies, the epileptiform activity could act as a catalyst that accelerates and worsens the maladaptive synaptic modifications that ultimately produce an imbalance between excitatory and inhibitory neurotransmission, which, in turn, rekindles the epileptogenic foci giving rise to a dangerous vicious circle [23].

Quite surprisingly, notwithstanding the wider choice of medications available and the progress of neurophysiology, neuropathology, and drug discovery in this research field, the percentage of epileptic patients that do not reach complete control of their symptoms even after an optimal therapeutic regimen (as indicated by the current guidelines) [24] did not change significantly [25]. This phenomenon may have something to share with the recent agreement of the scientific community on the proper name that should be given to the drugs actually administered to treat epileptic patients: ASMs. The conceptual difference with the previously (and sometimes still) accepted term “anti-epileptic drugs” is not trivial. In fact, the use of the expression “anti-seizure” instead of “anti-epileptic” efficaciously sums up the ability of these compounds to target mechanisms that enable the generation and propagation of seizures without posing an end to the physiopathological phenomena that underlie their onset and recurrence and ultimately make the brain, and hence the patient, epileptic [26].

This fundamental concept acquires even more paramount importance in the framework of neurodevelopmental epilepsies. In these cases, not only the well-being of the children in terms of seizure-freedom is at stake but also the correct development of a maturating brain and all the consequences that a premature blockade or an impairment of its delicate physiological milestones can determine.

Indeed, most ASMs are licensed for adult populations, and data on their use in children are often scarce. For this reason, concerns about ASMs’ compatibility with favorable cognitive and behavioral outcomes are one of the main concerns of pediatric epileptologists [27]. Moreover, to make things worse, it is extremely frequent for ASMs to somehow impact cognition since both monotherapeutic and polytherapeutic regimens can, by themselves, induce even serious cognitive impairment [28].

To give you a few examples: it has been known for decades that carbamazepine (CBZ, one of the most universally used ASMs) may not have a significant impact on cognition when administered in monotherapy, but it can cause significant cognitive and psychomotor disturbances when it is used as an add-on with a previously administered pharmacological agent [29]. Moreover, topiramate (TPM) is known for its ability to impair attention and verbal function [30].

On the other hand, it appears that some ASMs have fewer effects on cognitive capabilities, or none at all, but the issues and perplexities stemming from their use in a developing nervous system still stand.

In order to avoid and overcome the limitations associated with the use of “traditional” ASMs, recent efforts have been made to investigate the possibility of using pharmacological agents with mechanisms of action that explore new grounds in the field of epilepsy treatment.

### 2.1 Novel Therapeutic Approaches in Neurodevelopmental Pathologies

An interesting therapeutic opportunity in neurodevelopmental conditions lies in their “pre- determined” derangement from the physiological brain development, triggered in each case by specific pathogenic factors that are known, in most cases, and sometimes can be pharmacologically targeted.

Among the most studied diseases that fall into the aforementioned definition is tuberous sclerosis complex (TSC), a syndrome caused by the mutation of TSC1 and TSC2 genes. TSC is one of the most studied neurodevelopmental syndromes associated with epilepsy [31], and the approach to its pharmacological management clearly sums up how therapeutic strategies are evolving in these disorders.

Initially, as in most epileptic conditions, ASMs were administered to TSC patients only after the onset of epileptic seizures. Recently, considering that up to 90% of TSC patients eventually develop epilepsy, preemptive treatment with vigabatrin (VGB), an irreversible inhibitor of the GABA-aminotransferase, has been considered as a possible way to target “epileptogenesis” before it leads to spontaneous seizures in these patients [32, 33]. The reason why VGB is particularly efficacious in TSC is still partially unknown, but the mechanism of action of this drug, which results in an increase of GABA concentration in CNS, suggests that epileptogenesis in TSC may be dependent on impairment of GABAergic neurotransmission and thus VGB would recover the lost equilibrium between excitation and inhibition, reducing the risk and severity of epilepsy in TSC (Table **1**) [33, 34].

TSC is also characterized, at a molecular level, by the uncontrolled activation of the mTOR pathway resulting from the failure of its regulatory genes [35]. A recent class of drugs, the mTOR inhibitors, has been devised to block this pathogenetic mechanism, thus acting on the cause of the pathology and not on its consequences. Again, and not surprisingly, regularizing mTOR function seems to be beneficial also because it recovers the impairment of glutamatergic and GABAergic neurons, [36] regularizes the expression of ion channels and recovers synaptic plasticity [37, 38].

Interestingly, these drugs are not only useful in TSC but can find their place in several neurodevelopmental pathologies determined by mTOR hyperactivation (now collectively known as “mTORopathies”), such as focal cortical dysplasia (FCD), hemimegalencephaly, and ganglioglioma (Table **1**) [39].

As in the aforementioned example, similar therapeutic approaches acting on pathogenetic factors are being studied in other neurodevelopmental conditions, even though in not all these cases, clinical trials on patients are available yet.

For instance, Dravet syndrome, an epileptic encephalopathy mostly caused by mutations in the SCN1A gene, [40] is also characterized by immature GABAergic neurotransmission [41, 42], which could be involved in the generation of seizures and epileptogenesis in this disease. Interestingly, a novel and peculiar anticonvulsant that has been introduced as adjuvant therapy for this disorder, stiripentol, has the ability to positively modulate GABA_A_ receptors (GABA_A_Rs), even though the current potentiation is higher at α_3_ subunit-containing receptors [43]. Noteworthy, GABA_A_Rs containing α_3_ are especially expressed in the immature nervous system [44], which allows us to hypothesize that this drug acts more potently on immature receptors, thus compensating for their dysfunctional behavior in an adult brain.

### 2.2 The Case of “Bumetanide” and IGF1 in Chloride Homeostasis

The depolarizing (or “less hyperpolarizing”, according to some authors) effect of GABA-evoked currents in the developing brain has been a discovery that opened many new perspectives in physiology and pharmacology [45, 46]. In particular, many studies have described an immature and hence “less hyperpolarizing” GABAergic neurotransmission in several neurodevelopmental disorders with epilepsy, such as TSC, FCD, Dravet syndrome, and Rett syndrome [47-50].

This finding was frequently reported together with the dysregulation of a family of proteins, the cation-chloride cotransporters (CCCs), that are responsible for the regulation of intracellular chloride concentration. Specifically, the two major actors in this scenario are NKCC1, which is responsible for chloride import being frequently upregulated in neurodevelopmental diseases, and KCC2, which mediates chloride extrusion [51].

Starting from this observation, many authors proposed that reverting this alteration of GABAergic transmission and chloride homeostasis could greatly benefit the efficacy of inhibitory synaptic transmission in the aforementioned conditions, hence impacting both ictogenesis and cognitive outcomes [50].

A paradigmatic case in this scenario is that of bumetanide (BUME), a diuretic chloride importer NKCC1 antagonist [52]. By blocking NKCC1, this drug reduces the intracellular chloride concentration, thus favoring the inhibitory actions of GABAergic neurotransmission in physiopathological conditions where the normal chloride homeostasis is impaired [50, 53].

Although promising, the use of this drug came with some potential limitations and controversies. At first, it was reported that, at diuretic doses, the concentration of BUME that reached its target in the CNS was too low to be compatible with a significant effect [54] but, on the other hand, a recent clinical trial on neonates with seizures found an additional reduction in seizure burden attributable to BUME over phenobarbital without increased serious adverse effects [55].

Indeed, new clinical trials will shed more light on this important issue, but the search for alternative compounds that could selectively modulate chloride homeostasis is continuing. To this purpose, one strategy is to synthesize bumetanide derivatives with better pharmacological properties [56], such as better penetration through the blood-brain barrier and better NKCC1 selectivity, since BUME also affects NKCC2 (which is primarily expressed by epithelial cells of Henle’s loop) [56].

However, other studies demonstrated that the selective blockade of NKCC1 may not be the only available option to recover a physiological chloride equilibrium in neurodevelopmental pathologies. Indeed, there are conditions in which the unbalanced expression of the two transporters may be due to an insufficient function of KCC2. This is apparently the case of Rett syndrome, a condition arising mainly as a consequence of mutations in the MeCP2 gene [48]. In fact, knocking down MeCP2 in cultured mouse cortical neurons leads to a decreased KCC2 expression level and determines a delayed GABA functional switch [48]. Consequently, several compounds capable of enhancing KCC2 expression have been identified and tested both in culture, where they rescued electrophysiological and morphological abnormalities of RTT neurons, and in *MeCP2* mutant mice, in which they ameliorated disease-associated behavioral deficits [57]. In conclusion, regardless of the pathogenesis and the therapeutic strategy that is used to target the chloride homeostasis and GABAergic function impairment, it seems that restoring a correct balance of these important physiological mechanisms can have consequences that extend from the synaptic to the behavioral level (Table **1**) [50, 57, 58].

### 2.3 Cannabinoids and Synaptic Transmission

Cannabinoids are a class of compounds currently under the spotlight for their continuously increasing potential in the treatment of many neurological disorders. On the other hand, some aspects of their mechanism of action are not yet entirely elucidated, contributing to their limited safety and optimal clinical use [59]. While the effect of endocannabinoids (ECs) that bind to G protein-coupled receptors (CB1R and CB2R) has been well characterized, the extracts from the plant *Cannabis sativa* (more than 100 compounds) are not yet fully studied. This large group of compounds and their mechanisms of action are unclear, likely involving different and complex signaling pathways and receptors [60].

Indeed, two compounds in cannabis extract have been thoroughly investigated: ∆9- tetrahydrocannabinol (∆9-THC) and cannabidiol (CBD). While ∆9-THC possesses strong psychoactive action binding to CB1R and CB2R, CBD does not show any psychoactive effect, thus resulting in more tolerated and safer use in clinical practice [60]. In addition, another *Cannabis* derivative, cannabidivarin (CBDV), has been studied for its potential anti-seizures effect and anti- epileptic action both in animal models and humans [61].

However, it seems that these compounds can be helpful in the treatment of epilepsies associated with neurodevelopmental disorders. Indeed, FDA-approved prescription drugs containing CBD already exist and can be used for the treatment of Dravet syndrome, Lennox-Gastaut syndrome, and severe epileptic encephalopathies characterized by childhood-onset [62-66].

Furthermore, recent evidence suggests that new disorders may soon join this list. CBD is now used as add-on therapy for TSC-associated seizures [67] as TSC patients with refractory seizures showed a significant reduction in weekly seizure frequency (-48.8%) after 3 months of treatment with CBD in a clinical trial [68].

Additional promising results are coming from basic research, which could shed some light on the mechanism of action of these drugs. Notably, in a zebrafish model of TSC, the number of phosphor-S6-ribosomal protein-positive cells [69] was diminished after CBD treatment. This is very interesting since it suggests that this class of drugs may not only alleviate seizures but also influence a pathogenic factor of the disease itself, even though, at present, this remains a working hypothesis and more studies on animal models are needed to support this statement.

Moreover, cannabinoids (particularly CBD) are also capable of enhancing the function of GABA_A_Rs. CBD is able to potentiate GABA-evoked currents, especially at α2−containing receptors, and this current potentiation is independent of the classic benzodiazepine binding sites [70]. Additionally, this effect has also been observed in cortical tissues from Dravet syndrome patients and tuberal tissues from TSC patients [41].

Currently, CBD is being studied and tested in several other conditions, among which developmental and epileptic encephalopathies other than Dravet syndrome and Lennox-Gastaut syndrome are prominent. Indeed, patients with CDKL5 deficiency disorder and Aicardi, Dup15q and Doose syndromes, SYNGAP1 encephalopathy, and epilepsy with myoclonic absences may be involved in future clinical research (Table **1**) [71, 72].

### 2.4 Natural Substances as Potential Therapeutic Agents

In recent years, the idea that the canonical pharmacological treatment of epileptic disorders could be integrated with natural and/or dietary supplements has gained increasing attention and credit in the scientific community.

This interest stems at least partly from the demonstrated efficacy of the ketogenic diet in the treatment of refractory epilepsies and the fact that this dietary intervention can produce better results than standard pharmacological treatment in selected cases, such as that of GLUT-1 deficiency epilepsy. Indeed, the use of a ketogenic diet in this congenital metabolic disorder is recommended by current guidelines and can potentially alleviate some of the neurodevelopmental alterations that characterize this condition [73]. However, other promising “natural” interventions have been studied to date.

Short-chain fatty acids, such as acetate, propionate and butyrate, could modulate neurotransmission function by regulating brain neurochemistry. For instance, peripheral acetate administration can affect the hypothalamic glutamate-glutamine coupling cycle, determining changes in GABA metabolism [74]. Furthermore, propionate and butyrate influence the transcription of several genes that control the production and degradation of serotonin and catecholamines (*e.g*., tyrosine hydroxylase) [75], which could also have an impact on seizure control [76].

Other plausible supplements implicated in seizure control are vitamins and antioxidants, which frequently share similar mechanisms of action. The rationale behind the potential usefulness of these compounds in epilepsy and, specifically, in developmental syndromes is that oxidative stress could be a common pathological hallmark of both adult epilepsies, such as temporal lobe epilepsy (TLE) [77] and developmental epileptic syndromes, such as FCDIIb and TSC [78], likely triggering harmful phenomena, such as iron accumulation and apoptosis [78, 79]. In the first category, the effect of the group of chemicals that commonly fall into the “vitamin E” group is well-characterized. Among these substances, α-Tocopherol is one of the most biologically active in humans [80]. The antioxidant capacities of these molecules could justify why their use in epileptic and neurodegenerative disorders is encouraged and is possibly beneficial, even though evidence in humans is still inconclusive. On the other hand, it has been hypothesized that scavenging reactive oxygen species during epileptogenesis could slow down and potentially prevent epilepsy onset [81]. Beyond this, recent research pointed out that α-tocopherol can also decrease the levels of inflammatory mediators released post-status epilepticus, potentially inhibiting epileptogenesis progression [82, 83].

Other vitamins, such as vitamin B6 and vitamin C, possess interesting anti-seizure effects, of which we only report some of the most significant. For example, vitamin B6 derivatives (as pyridoxamine 5’-phosphate and pyridoxal 5’-phosphate) are involved in the conversion of glutamate to GABA [84] and their deficiency could result in excess glutamatergic excitation. Accordingly, pyridoxal 5’-phosphate serum levels are often low in epilepsy patients [85,86]. On the other hand, vitamin C is implicated in analogous anti-seizure mechanisms due to its antioxidant and neuromodulatory effects [87].

The list of the antioxidant substances and “natural integrators” could be far longer, and the accurate description of each one of these is beyond the scope of this review. However, even though evidence supporting the use of these substances is abundant, it should be underlined that, at present, the translational potential of most of these agents still needs to be tested through randomized clinical trials involving epileptic patients.

A last mention should be reserved for endogenous agents which could exert anti-seizure or anti- epileptogenic actions. Erythropoietin, the cytokine that stimulates red blood cells production, can promote neurogenesis and neuroprotection in epilepsy [88]. It was also demonstrated that it could potentiate GABAergic neurotransmission both by increasing GABA_A_R currents amplitude [89] and by attenuating loss of potassium-chloride cotransporters in prenatal brain injuries, which could limit the impact of these events in subsequent brain maturation [90]. However, concerning erythropoietin as a potential ASM, we could act by potentiating the beneficial signaling pathways that it can trigger rather than intervening with the synthetic substance, as well as for other endogenous agents that recently attracted the attention of the epileptologists (*i.e*., brain-derived neurotrophic factor, BDNF) [91]. Future research is needed to elucidate all these recent evidences for new therapeutic approaches for refractory epilepsies.

## 3 NEURODEGENERATION AND EPILEPSY: A SELF-POWERING LOOP

Several preclinical and clinical studies suggest a close correlation between epilepsy and cognitive decline [92]. Nevertheless, their relationship remains controversial, and whether these disorders share some underlying pathophysiologic mechanisms or whether one is the collateral manifestation of the other is still a matter of debate. In this respect, the evidence of seizures in the prodromal phase of several neurodegenerative disorders also leads to the hypothesis that seizures can be considered “a part of the phenotypic spectrum at any stages of the neurodegenerative diseases” [93].

Many studies have addressed the risk of seizures among patients with AD, and it is well known that patients with AD have a 10 times higher risk of developing epilepsy [94]. Moreover, evidence in patients with mild cognitive impairment or AD suggests that seizures may accelerate the onset of cognitive decline [95, 96]. On the other hand, the risk of cognitive impairment among people with epilepsy has been less investigated. However, clinical evidence indicates that approximately 50% of epilepsy patients experience cognitive deficits [97, 98] and that people with epilepsy have a threefold increased risk of dementia compared with the general population, with the risk particularly enhanced when the onset of epilepsy is late adult life [92, 99, 100]. In this respect, studies indicate that patients with late-onset epilepsy of unknown aetiology (LOEU) are more likely to develop dementia [101] and show a significant cognitive decline at 12 months of follow-up [102].

Moreover, it has been reported that patients with LOEU have pathological CSF amyloid-β (Aβ) 1-42 and t-tau levels compared to healthy individuals [103, 104], with Aβ deposition increasing their risk of developing cognitive decline over the decades following seizure onset. This evidence clearly suggests that Aβ pathology might lead to both epileptogenic alterations and cognitive impairment. In this respect, in a preclinical study, the authors reported that acute exposure of mouse hippocampal slices to Aβ1-42 oligomers lowered the epileptic threshold and impaired synaptic plasticity in the dentate gyrus (DG) of the hippocampus with a mechanism dependent on D1 dopamine receptor stimulation signaling, in which AMPAR subunits rearrangements were also involved [103]. However, cognitive impairment may also be part of the epileptogenic process.

In fact, Aβ is pro-epileptogenic at the oligomer stage, and its accumulation fosters network hyperexcitability, which in turn enhances amyloid deposition, in a vicious loop, finally resulting in neuronal loss, impaired synaptic plasticity and homeostasis, and network remodeling of neuronal circuits [92, 105]. In this respect, it has been recently proposed that LOEU, epileptic prodromal AD and seizures in AD are all possible clinical correlates of an Aβ-driven continuum [92].

All these evidences highlight the possibility of speculating similar associations between epilepsy and other neurodegenerative proteins, such as, for instance α-synuclein. However, to date, very limited investigations exist on the impact of α-synuclein-mediated neurodegeneration in epileptogenesis, despite some clinical studies have reported an upregulated α-synuclein expression in epileptic patients, thus highlighting the possibility of α-synuclein-mediated neurodegeneration in epilepsy [106, 107]. Although the neurodegenerative role of α-synuclein is well established, it is still debatable whether hyperexcitability can be considered a key feature of α-synucleinopathies and Lewy body dementias (LBDs). Indeed, although both preclinical and clinical studies suggest a possible correlation between α-synuclein and epileptic seizures [106-109], whether hyperexcitability can represent a consistent feature of early α-synucleinopathy is still unclear [110-112].

All these findings have increased the attention on neurodegenerative proteins as possible targets to develop promising therapeutic approaches against epilepsy. On the other hand, it is also plausible that a targeted intervention to reduce abnormal network hyperexcitability might constitute a therapeutic strategy to delay the onset of neurodegenerative changes and consequent cognitive decline in patients. In this respect, given the experimental evidence and models supporting Aβ epileptogenic potential [92], several ASMs have been widely explored for their ability to limit Aβ epileptogenic potential [113, 114] and for their ability to improve cognitive deficits related to Aβ pathology. In this respect, while data regarding the eventual benefits of first-generation ASMs are somewhat controversial [92], more promising results have been obtained with second- and third-generation drugs. Indeed, CBZ has been reported to significantly reduce spontaneous electrographic epileptic discharges in APP/PS1 transgenic mice and to improve cognitive impairment in APP/PS1ΔE mice by reducing Aβ plaque burden [115]. Moreover, CBZ is neuroprotective against exposure of hippocampal neurons to Aβ peptide *in vitro* with a mechanism involving the stabilization of free calcium intracellular concentrations [116].

In the same *in vivo* models, valproic acid (VPA) has also been shown to limit the epileptiform activity in a dose-dependent (but not long-lasting after treatment discontinuation) manner [117, 118] and reduce Aβ plaque deposition in both the cortex and the hippocampus [119]. VPA has also been reported to increase the survival of hippocampal neurons exposed to Aβ_25-35_ fibrils [120]. Interestingly it has also been reported that administration of VPA increases histone acetylation at promoters of genes involved in memory formation and plasticity [118], such as BDNF, NR2A, and CDK5 [121-124]. Effects similar to those of CBZ have also been observed for lamotrigine (LTG), able to suppress cortical epileptic activity, prevent dendritic spines loss, and attenuate deficits of synaptic plasticity in APP/PS1 mice, with a mechanism involving the reduction of Aβ plaques possibly through modulation of AβPP cleavage by β-secretase [125, 126]. Moreover, enhanced levels of BDNF and NGF were found in the brain of transgenic mice chronically treated with LTG [126], thus suggesting that this drug might counteract amyloidosis through multiple mechanisms, that include modulation of abnormal network activity, as well as reduction of Aβ production and upregulation of neurotrophic factors deeply involved in synaptic plasticity, learning and memory processes (Table **1**).

Perhaps, the most promising second-generation ASM is levetiracetam (LEV), which has been shown to reduce cortico-hippocampal hyperexcitability and seizures in different experimental models, namely APP/PS1 [127], 3×Tg [114] and hAPP mice [128]. Interestingly, LEV has a unique mechanism of action compared to other ASMs, that involves the binding to presynaptic vesicle protein 2A [129], which may underlie its protective effect against seizure [130]. Moreover, LEV inhibits calcium release from intraneuronal stores [131, 132] and N-type calcium channels.

Since calcium dyshomeostasis is accelerated by Aβ oligomers [133, 134] and represents a very early phenomenon in the process of amyloidosis, targeting calcium dyshomeostasis can likely constitute a tool to reduce hyperexcitability and Aβ deposition [134, 135]. Similar to VPA, LEV also reduces Aβ plaque deposition in both the cortex and hippocampus [119] of APP/PS1 mice. However, a direct effect of this drug on Aβ turnover has not been confirmed in similar experimental models.

LEV has also been shown to restore synaptic deficits [128] and to prevent neurodegeneration caused by exposure of hippocampal neurons to Aβ oligomers [136]. This neuroprotective action of LEV likely relies on its ability to prevent Aβ-induced glutamate release at extrasynaptic sites [137], thus limiting excitotoxicity (Table **1**).

Clinical evaluations of LEV in AD have been limited to people with overt clinical seizures and to people with mild cognitive impairment at risk of developing AD, in which LEV improved memory function potentially through changes in hippocampal activation [138]. A randomized, double-blind, placebo-controlled crossover study (ILiAD) is currently open to evaluate whether treatment with LEV is more effective than a placebo in restoring or preserving memory function in people with AD [138]. If this study demonstrates at least the stabilization of memory function, the next step could be to study whether this drug may be useful in combination with other disease-modifying therapies for AD. It appears that achieving an appropriate aetiological classification and accurate risk stratification of cognitive decline is a key step in identifying patients eligible for therapeutic interventions at early prodromal stages before Aβ triggers neurodegeneration. In this time-critical window, disease-modifying treatment is likely the most beneficial [92].

Although limited, overall, these experimental evidences suggest that ASMs can exert neuroprotective activity and slow neurodegeneration and loss of synaptic plasticity associated with Aβ pathology. In this respect, it is possible to speculate that ASMs might improve cognitive deficits in AD. The answer is not simple because improving cognition may require a delicate balance between reducing synaptic hyperexcitability and inducing synaptic depression. Indeed, some ASMs (such as benzodiazepines, CBZ, eslicarbazepine, oxcarbazepine, phenobarbitone, phenytoin, primidone, tiagabine (TGB), TPM, VPA, VGB, and zonisamide (ZNS)) also have been demonstrated to possess cognitive side effects [93].

Aside from the link between epilepsy and neurodegeneration in models of amyloidosis, studies in different models have identified a plethora of neurodegenerative changes that occur during epilepsy that are also involved in the pathogenesis of different neurological disorders, including processes of neuroinflammation [139], mitochondria dysfunction, oxidative stress, changes in the expression, and function of glutamate and GABA receptors, and fiber sprouting, which may be potential targets for pharmacological approaches [140-142]. In particular, activation of the mitochondrial permeability transition and generation of free radicals can be a consequence of glutamate-excitotoxicity that constitutes a common pathogenetic pathway in a variety of neurodegenerative diseases with distinct etiologies [17, 143]. Reduced activity of complex I of the mitochondrial respiratory chain has been implicated in several neurological disorders, such as PD and Down syndrome [144]. Indeed, inhibition of mitochondrial respiratory complex I with rotenone is considered a model to reproduce PD [141]. Among the various ASMs, CBZ and ZNS have been found to exert a neuroprotective action against rotenone-induced striatal neuronal dysfunction [145, 146] with a mechanism involving activation of GABA_A_Rs and consequent enhancement of GABA-mediated inhibition. Apart from the interaction with GABA_A_R, the ZNS neuroprotective action may be due to its antioxidant properties [146], the inhibition of carbonic anhydrase, and consequent changes in neuronal pH and activity [147]. It is interesting to note that the neuroprotective action of ZNS is achieved at doses lower than those used for anticonvulsant therapy [146]. A novel strategy to reduce oxidative stress, as well as neuroinflammation and excitotoxicity in neurodegenerative disorders, is represented by CBD. Indeed, *in vitro* studies demonstrate that treatment with CBD counteracts Aβ-induced neurotoxicity [148] and tau hyperphosphorylation [149] while promoting hippocampal neurogenesis and neuronal survival [150, 151]. In this respect, CBD administration is able to reduce Aβ formation [152] and ameliorate Aβ-induced memory impairment in rodents [153-155]. Other *in vitro* studies indicate that CBD exerts its neuroprotective effect not only by stimulating the clearance of intraneuronal Aβ [156] but also by acting on signaling pathways involved with proteostasis [157], downregulating genes encoding for secretases and kinases involved in the development of AD and upregulating the ubiquitin systems [158]. Moreover, in models of AD, CBD has been demonstrated to reduce neuroinflammation, reactive oxygen species generation, and mitochondrial dysfunction by interacting with the Wnt/β-catenin pathway and peroxisome proliferator-activated receptor-γ (Table **1**) [152, 159].

Although much evidence points to a neuroprotective role of ASMs, it must be taken into consideration that the concept of neuroprotection should not be limited to the prevention of neuronal death only but rather includes prevention against the neuronal and network dysfunctions that occur during the development of chronic epilepsy and cognitive decline [160,161]. Indeed, since the epileptic activity is sustained at the network level, it is likely that a wide loss of neurons hinders epilepsy [162], and endogenous neuroprotective pathways involved in axonal rearrangement and enhancement of synaptic transmission may result in proepileptogenic [163].

## 4 BRAIN ISCHEMIA AND EPILEPSY: FOCUS ON NEUROPROTECTION

It is currently well established that synaptic and cellular events induced by cerebral ischemia share physiopathological mechanisms with those triggered by the abnormal neuronal discharge induced by epilepsy [164, 165]. Indeed, the epileptic discharge keeps the hyperactive neuron in a sustained depolarized state resulting in an impaired energy supply, similar to what occurs during the ischemic process. In particular, the failure of the Na^+^/K^+^ pump leads to the rundown of ionic gradient and consequent further depolarization of the neuron, loss of ionic homeostasis, and a massive release of excitatory amino acids into the extracellular space. Since these processes involve both pre- and post-synaptic terminals, as well as glial cells, the reuptake of excitatory neurotransmitters is impaired with sustained activation of ionotropic and metabotropic glutamate receptors, further contributing to calcium dyshomeostasis, resulting in cellular damage and even death. ASMs are able to reduce neuron excitability by acting on different molecular targets, such as voltage-gated sodium and calcium channels, GABA or glutamate receptors [18]. Therefore, it is not surprising that ASMs may be able to reduce ischemic injury [165]. Several ASMs have been tested in multiple models of global or focal ischemia with encouraging results. The classic mechanism of action of ASMs implies that these drugs are able to counteract hyperexcitability by either decreasing excitatory transmission or enhancing neuronal inhibition [18]. Accordingly, it has been demonstrated that both GABAergic drugs, such as TGB and VGB, and anti-glutamatergic drugs, such as LTG, are neuroprotective against *in vitro* ischemia [166, 167]. In this respect, an interesting study compared the neuroprotective effect toward *in vitro* ischemia in corticostriatal brain slices of two classic (CBZ and VPA) and two new generations (TPM and LEV) ASMs [168]. The authors found that CBZ, VPA, and TPM but not LEV exerted neuroprotective effects, achieved by concomitant inhibition of fast Na^+^ and L-type Ca^2+^ conductances. In contrast, presynaptic inhibition of glutamate transmission did not seem to be involved. Interestingly, the neuroprotective effect of CBZ and TPM was achieved at concentrations comparable to the therapeutic levels found in cerebral spinal fluid and serum of epileptic patients [169, 170] and able to inhibit fast Na^+^ and high voltage-activated (HVA) Ca^2+^ currents, the concentrations of VPA required, although much higher than those reported to be safe in these patients [171], failed to affect both types of conductances. It is likely that the neuroprotective action of VPA relied on its capability to modulate caspase activity [172] and probably involved apoptotic mechanisms [173].

Interestingly, it has been reported that VPA is able to recover hippocampal synaptic plasticity and improve impairment in learning and memory as well as in spatial cognitive performance in different models of *in vivo* ischemia [174-176].

It has been widely reported that ASMs may negatively affect cognitive functions by affecting synaptic plasticity and network development in a dose-dependent manner [27, 177-180]. For this reason, when exploring the neuroprotective action of ASMs, it is of fundamental importance to evaluate their effect on physiologic synaptic transmission and plasticity. In this view, the ideal condition is to achieve neuroprotection at a concentration of ASM that *per se* does not alter physiological synaptic transmission and plasticity. In this respect, both lacosamide (LCM) and perampanel (PER) have been demonstrated to provide neuroprotection to both hippocampal and striatal neurons from oxygen and glucose deprivation (OGD) at concentrations that do not impair basal transmission and physiological long-term potentiation (LTP) [181, 182]. Differently from classic ASMs, LCM does not affect the fast inactivation of voltage-gated Na^+^ channels but rather modulates them by selectively enhancing their slow inactivation kinetic and consequently increasing the probability that these channels are in the slow inactivated state [183].

Regarding the neuroprotective effect of PER, it is interesting to note that this is achieved not only at concentrations unable to affect the physiologic synaptic transmission but also at lower concentrations than those required for its anti-seizure action [182]. This might be due to the selective action of PER on GluA1 subunits of AMPARs. Indeed, OGD increases the expression of GluA1 subunits [182], and consequently, the presence of GluA2-lacking Ca^2+^-permeable AMPARs at the synapses, so it is likely that the enhanced expression of GluA1 determines the increased sensitivity of neurons to the action of low concentrations of PER. Thus, the selective inhibition of GluA1 subunits by PER determines neuroprotection while rescuing the physiological Ca^2+^-impermeable AMPARs at synapses. Another possibility is that PER exerts its beneficial effect by acting on non-synaptic AMPARs or on different, still unveiled, neuroprotective pathways. PER has also been demonstrated to ameliorate the post-stroke motor function and the spatial working memory in a rat model of transient middle cerebral artery occlusion through possible anti-inflammatory and antioxidant mechanisms [184] and to reduce brain infarct volume and neuronal apoptosis following focal cerebral ischemia in rats [185].

Furthermore, at the same concentrations, PER has been shown to inhibit ischemic LTP [182], a form of pathologic long-term potentiation triggered in the hippocampus by acute OGD and not linked to associative learning and memory formation [186]. The ASM ZNS, able to prevent ischemic damage in animal models [187-189], also showed neuroprotective effects against ischemic LTP at doses lower than those used for anticonvulsant therapy [190] and that were unable to affect both hippocampal and cortico-striatal excitatory synaptic transmission [146, 190] as well as physiologic LTP [190]. ZNS has a specific pharmacological profile and antioxidant properties [147]. A reduction of NO is one possible reason for the neuroprotective properties of ZNS. Indeed, the NO/cGMP/PKG pathway exerts a critical role in the induction of the hippocampal ischemic LTP, and ZNS reduces this pathological plasticity by modulating this biochemical pathway [190].

Robust anti-oxidant and anti-inflammatory properties have also been demonstrated by CBD, which emerges as a solid candidate to exert neuroprotection in animal models of adult [191, 192] and post-natal brain ischemia [193-197]. Among the mechanisms through which CBD exerts neuroprotection, a reduction in glutamate release, an increase in extracellular adenosine levels, and inhibition of NFkB have been demonstrated [194, 198]. Indeed, CBD has been reported to reduce excitotoxicity and apoptotic processes, as well as astrogliosis and neuroinflammation in experimental ischemia models [199]. Moreover, CBD increases the levels of BDNF in the hippocampal CA1 of rats submitted to transient global cerebral ischemia, thus attenuating memory deficits and degeneration of dendritic spines (Table **1**) [200].

Overall, preclinical data strongly suggest a neuroprotective role exerted by several ASMs highlighting the possibility of future therapeutic strategies to prevent or delay the neurodegenerative process. At present, however, very few attempts have been made in humans. In this respect, among new-generation ASMs, LEV has been reported to improve cognitive functions in amnestic mild cognitive impairment and AD patients [138, 201], while improvements in partial cognitive performance have been reported for PER, LCM, and oxcarbazepine [102, 202, 203]. Adverse cognitive effects of antiseizure pharmacotherapy must also be taken into consideration as possible side effects. Indeed, some ASMs (*e.g*., TPM, ZNS) show a more severe side effect profile than others (*e.g*., LTG, LEV). In this respect, several risk factors have been established. In particular, it has been reported that a higher drug load and polytherapy increase the risk of adverse cognitive side effects [178].

Moreover, other factors, such as age, seizure frequency and type, may contribute. In conclusion, despite the promising results from preclinical studies, further clinical investigation is necessary to translate these findings to human patients. Thus, neuroprotection remains an important challenge for the future.

## CONCLUSION

**Fig. (1):**
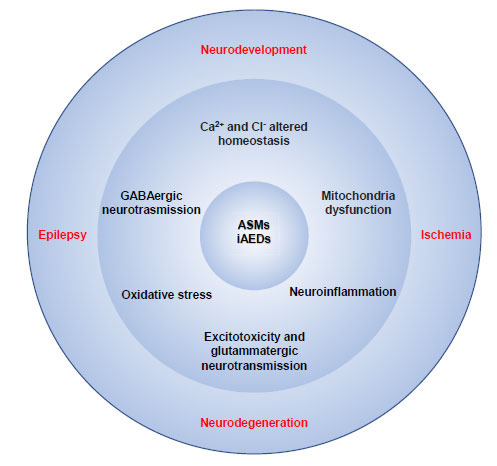
Multiple molecular mechanisms intertwining epilepsy with neurological and neurodevelopmental patologies can constitute the target of traditional and innovative ASMs and the substrate for the development of new classes of multitarget drugs, new classes of multitarget drugs, that can potentially constitute “innovative anti-epileptic drugs” (iAEDs) with disease-modifying effects toward precision therapy.

By reviewing the most recent literature regarding ASMs, it becomes clear that traditional drugs and their mechanisms of action that target the symptom and not the cause of the disease will probably have a progressively minor role in the treatment of epileptic disorders and their comorbidities. From the research in this field progresses, it appears clear that the concept of “treatment” itself is changing, and it is gradually shifting from a vision in which seizure control was the ultimate and most important goal to one where global care of the disease and its comorbidities may be obtained at the same time if the right physiopathological factors are targeted pharmacologically. From all the evidences discussed here, it is possible to argue that, despite the different mechanisms of action, the ASMs (especially multitarget ASMs) are promising in terms of neuroprotection and recovery of physiological neurodevelopment (Fig. **1**). This opens up the possibility of a new approach to the production and screening of new classes of drugs that can act specifically on the altered neurobiological mechanisms contributing to the development of both epilepsy and neurodegeneration, thus preventing or modifying the reorganizations of neuronal circuits that produce a lowered seizure threshold and consequently cognitive impairment.

Furthermore, the same strategy can be applied to the field of neurodevelopmental epilepsies, in which new drugs will aim not exclusively to reduce symptoms but also to target etiopathogenic factors as the basis of the disease. This approach will allow to re-establish the physiological development of the nervous system, at the same time slowing down, or possibly blocking, epileptogenesis and onset of neuropsychological comorbidities.

These new classes of drugs, specifically targeting the underlying physiopathological mechanisms of epilepsy and altering its course, can potentially constitute “nonconventional anti-epileptic drugs” with disease-modifying effects.

## Figures and Tables

**Table 1:** ASMs and experimental models of specific neurological pathologies in which a neuroprotective effect has been demonstrated.

**ASM**	**Experimental Model**	**Neurological Disease**	**Neuroprotective/Cognitive Effects**	**References**
Carbamazepine	APP/PS1ΔE mice; Hippocampal neurons in culture exposed to Aβ fibrils	Cerebral amyloid-β pathology, Alzheimer’s disease	Improved cognitive impairment in mice by reducing Aβ plaque burden; survival of cultured hippocampal neurons exposed ^to Aβ^_^25-35^_^fibrils^	[115, 116]
	Cortico-striatal brain slices exposed to rotenone	Parkinson’s disease	Neuroprotection of striatal neurons against rotenone-induced dysfunction *via* activation of ^GABA^A ^receptors^	[145]
	Cortico-striatal brain slices exposed to OGD	Brain ischemia	Neuroprotection at micromolar concentrations by reduction of L-type Ca^2+^ currents	[168]
Valproate	APP/PS1 and 3xTg-AD transgenic mice	Cerebral amyloid-β pathology, Alzheimer’s disease	Reversal of memory impairment;	[114, 116, 118, 120]
	APPswe/PS1dE9 transgenic mice	-	Increased acetylation of histone H3 at promoters of *BDNF*, *CDK5* and *NR2A* genes;	
	Hippocampal neurons in culture exposed to Aβ fibrils;	-	reduction of Aβ oligomers and deposition; increased survival of hippocampal neurons ^exposed to Aβ^25-35 ^fibrils^	
	7PA2 cells stably transfected with human APP	-	-	
	Cortico-striatal brain slices exposed to OGD	Brain ischemia	Concomitant inhibition of fast Na^+^ and L-type Ca^2+^ conductances	[168]
Lamotrigine	APP/PS1 transgenic mice	Cerebral amyloid-β pathology, Alzheimer’s disease	Reduced Aβ cleavage Reduced number and size of Aβ plaques; Increased expression of NGF and BDNF	[125, 126]
Levetiracetam	-	-	Recovery of synaptic and cognitive functions	[114, 119, 127-132, 136, 137]
	HEK 293T cells	-	Normalization of synaptic protein levels	
	Hippocampal neurons in culture exposed to Aβ oligomers; hAPPJ20 transgenic mice	Cerebral amyloid-β pathology, Alzheimer’s disease	Reduction of Aβ plaque burden *in vivo* Prevention of Aβ_25-35_ -induced neurodegeneration	
	APP/PS1 or 3×Tg mice	-	Neuroprotection through binding to presynaptic vesicle protein 2A	
	APPswe/PS1dE9 transgenic mice	-	Inhibition of calcium release from intraneuronal stores	
	-	-	Prevention of Aβ-induced glutamate release at extrasynaptic sites	
Zonisamide	Cortico-striatal brain slices exposed to rotenone	Parkinson’s disease	Neuroprotection of striatal neurons against rotenone-induced dysfunction *via* activation of ^GABA^A^R;^ antioxidant properties	[146, 147]
	-	-	Changes in neuronal pH and activity through inhibition of carbonic anhydrase	
	CA1 hippocampal slices exposed to OGD	Brain ischemia Focal cerebral ischemia	Neuroprotective effects against iLTP by modulation of NO/cGMP/PKG pathway	[147, 188-191]
	Carotid ligation in neonatal rats	-	Reduction of hypoxic-ischemic brain damage	
	-	Global forebrain ischemia	-	
	Rat middle cerebral artery occlusion-reperfusion	-	Reduced cerebral damage in and neurological deficit induced in the cortical and subcortical regions by transient ischemia	
	Carotid arteries occlusion in gerbils	-	-	
Lacosamide	Corticostriatal and hippocampal slices exposed to OGD	Brain ischemia	Neuroprotection of both striatal and pyramidal neurons from energy metabolism failure during OGD	[181]
Perampanel	Corticostriatal and hippocampal slices exposed to OGD	Brain ischemia Focal cerebral ischemia	Neuroprotection of both striatal and pyramidal neurons from energy metabolism failure during OGD. Neuroprotective effects against iLTP by selective inhibition of GluA1 subunits and rescue of the physiological Ca^2+^-impermeable AMPARs at synapses improvement of post-stroke motor functions and spatial working memory through anti-inflammatory and antioxidant mechanisms involving the activation of phosphatidylinositol 3-kinase (PI3K)/Akt pathways.	[182, 184, 185]
	Transient middle cerebral artery occlusion (MCAO) model			
Cannabidiol	β-amyloid-induced toxicity in PC12 cells	-	Protection from apoptosis *via* downregulation of caspase-3 expressions, reduction of ROS, intracellular Ca^2+^ levels and lipid peroxidation. Downregulation of genes coding for the genes coding for kinases responsible for aberrant tau phosphorylation (GSK3β, CDK5, DYRK1A, CAMK2A, MAPK1, MAPK12, and MAPK14) and for the secretases involved in Aβ generation (β-(BACE-1) and γ-secretases; increased expression of genes coding for members of the ubiquitin system; upregulation of PI3K/Akt signaling pathway. Reduction of intracellular NO level and NF-kB activation *via* inhibition of iNOS and p38 MAPK phosphorylation. APP protein ubiquitination and reduced Aβ production and neuroinflammation *via* PPARγ activation. Degradation and removal of preformed Aβ aggregates. Prevention of cognitive impairment and reduction ^of Aβ^40 ^levels.^	[148-150, 152-159]
	Mesenchymal Stem Cells	-		
	Human neuroblastoma SHSY5Y^APP+^ cells MC65 human neuron-like cell line engineered to produce Aβ aggregates	Proteinopathies (AD, PD, HD, MS) cerebral amyloid-β pathology, Alzheimer’s disease		
	C57/Bl6 AD mice	-		
	APPswe/PS1ΔE9 double transgenic AD male mice	-		
	AβPP/PS1 mice	-		
	Forebrain slices from newborn mice exposed to OGD MCAO in mice	-	Protection from apoptosis through reduction of caspase-9 levels; reduction of glutamate and IL-6 concentration; inhibition TNFalpha, COX-2, and iNOS expression. These effects are mainly mediated by CB2 and adenosine A2A receptors.	[191-194, 196, 197]
	Temporary occlusion of carotid arteries plus hypoxia in newborn pigs	Brain ischemia	Reduction of the infarct volume and neuroprotection through serotonergic 5-HT1A receptor-dependent increased cerebral blood flow to the cortex	
	Hypoxia after carotid artery electrocoagulation	-	Recovery of neurobehavioral functions	
	Oocytes transplanted with human tissues from TSC and Dravet patients	Dravet syndrome Lennox-Gastaut	Increase of GABAergic currents Immunosuppressive effects	[41, 62-66, 70]
	Clinical trials	TSC	Epigenetic mechanisms	
Vigabatrin	Clinical trial	TSC	Increase of GABA concentration in CNS	[33, 34]
mTOR inhibitors	Rat hippocampal neurons in culture	TSC, FCD, ganglioglioma	Recovery of the impairment of glutamatergic and GABAergic neurons. Regularization of the expression of ion channels and synaptic plasticity	[36-38]
Bumetanide and its derivatives	Clinical trial Xenopus oocytes	Neonatal seizures TSC Rett syndrome	Blockade of chloride transporter NKCC1	[42, 53, 55, 56]

## LIST OF ABBREVIATIONS

AD: Alzheimer's Disease
CNS: Central Nervous System
HD: Huntington’s Disease
MS: Multiple Sclerosis
ADHD: Attention-deficit/Hyperactivity Disorder
ASD: Autism Spectrum Disorder
TSC: Tuberous Sclerosis Complex
TLE: Temporal Lobe Epilepsy
DG: Dentate Gyrus
LBDs: Lewy Body Dementias
VPA: Valproic Acid
HVA: High Voltage-Activated

## References

[1] Beghi E. (2020). The epidemiology of epilepsy.. Neuroepidemiology.

[2] Austin J.K., Caplan R. (2007). Behavioral and psychiatric comorbidities in pediatric epilepsy: Toward an integrative model.. Epilepsia.

[3] Kanner A.M. (2016). Psychiatric comorbidities in epilepsy: Should they be considered in the classification of epileptic disorders?. Epilepsy Behav.

[4] Reilly C., Atkinson P., Das K.B., Chin R.F.M.C., Aylett S.E., Burch V., Gillberg C., Scott R.C., Neville B.G.R. (2014). Neurobehavioral comorbidities in children with active epilepsy: A population-based study.. Pediatrics.

[5] Bertelsen E.N., Larsen J.T., Petersen L., Christensen J., Dalsgaard S. (2016). Childhood epilepsy, febrile seizures, and subsequent risk of ADHD.. Pediatrics.

[6] Keezer M.R., Sisodiya S.M., Sander J.W. (2016). Comorbidities of epilepsy: Current concepts and future perspectives.. Lancet Neurol..

[7] Seidenberg M., Pulsipher D.T., Hermann B. (2009). Association of epilepsy and comorbid conditions.. Future Neurol..

[8] Colmers P.L.W., Maguire J. (2020). Network dysfunction in comorbid psychiatric illnesses and epilepsy.. Epilepsy Curr..

[9] Lignani G., Baldelli P., Marra V. (2020). Homeostatic plasticity in epilepsy.. Front. Cell. Neurosci..

[10] Powell K.L., Lukasiuk K., O’Brien T.J., Pitkänen A. (2014). Are alterations in transmitter receptor and ion channel expression responsible for epilepsies?. Adv. Exp. Med. Biol..

[11] Rao V.R., Lowenstein D.H. (2015). Epilepsy.. Curr. Biol..

[12] Pizzo L., Jensen M., Polyak A., Rosenfeld J.A., Mannik K., Krishnan A., McCready E., Pichon O., Le Caignec C., Van Dijck A., Pope K., Voorhoeve E., Yoon J., Stankiewicz P., Cheung S.W., Pazuchanics D., Huber E., Kumar V., Kember R.L., Mari F., Curró A., Castiglia L., Galesi O., Avola E., Mattina T., Fichera M., Mandarà L., Vincent M., Nizon M., Mercier S., Bénéteau C., Blesson S., Martin-Coignard D., Mosca-Boidron A.L., Caberg J.H., Bucan M., Zeesman S., Nowaczyk M.J.M., Lefebvre M., Faivre L., Callier P., Skinner C., Keren B., Perrine C., Prontera P., Marle N., Renieri A., Reymond A., Kooy R.F., Isidor B., Schwartz C., Romano C., Sistermans E., Amor D.J., Andrieux J., Girirajan S. (2019). Rare variants in the genetic background modulate cognitive and developmental phenotypes in individuals carrying disease-associated variants.. Genet. Med..

[13] Perucca P., Bahlo M., Berkovic S.F. (2020). The genetics of epilepsy.. Annu. Rev. Genomics Hum. Genet..

[14] Rho J.M., White H.S. (2018). Brief history of anti‐seizure drug development.. Epilepsia Open.

[15] Sills G.J., Rogawski M.A. (2020). Mechanisms of action of currently used antiseizure drugs.. Neuropharmacology.

[16] Löscher W. (2021). Single-target versus multi-target drugs versus combinations of drugs with multiple targets: Preclinical and clinical evidence for the treatment or prevention of epilepsy.. Front. Pharmacol..

[17] Calabresi P., Picconi B., Saulle E., Centonze D., Hainsworth A.H., Bernardi G. (2000). Is pharmacological neuroprotection dependent on reduced glutamate release?. Stroke.

[18] Rogawski M.A., Löscher W. (2004). The neurobiology of antiepileptic drugs.. Nat. Rev. Neurosci..

[19] Pitkänen A., Sutula T.P. (2002). Is epilepsy a progressive disorder? Prospects for new therapeutic approaches in temporal-lobe epilepsy.. Lancet Neurol..

[20] Blennow K., de Leon M.J., Zetterberg H. (2006). Alzheimer’s disease.. Lancet.

[21] Kapoor R., Furby J., Hayton T., Smith K.J., Altmann D.R., Brenner R., Chataway J., Hughes R.A.C., Miller D.H. (2010). Lamotrigine for neuroprotection in secondary progressive multiple sclerosis: a randomised, double-blind, placebo-controlled, parallel-group trial.. Lancet Neurol..

[22] Schapira A.H.V., Olanow C.W., Greenamyre J.T., Bezard E. (2014). Slowing of neurodegeneration in Parkinson’s disease and Huntington’s disease: future therapeutic perspectives.. Lancet.

[23] Brooks-Kayal A. (2011). Molecular mechanisms of cognitive and behavioral comorbidities of epilepsy in children.. Epilepsia.

[24] Kwan P., Schachter S.C., Brodie M.J. (2011). Drug-resistant epilepsy.. N. Engl. J. Med..

[25] Golyala A., Kwan P. (2017). Drug development for refractory epilepsy: The past 25 years and beyond.. Seizure.

[26] Fisher R.S., Acevedo C., Arzimanoglou A., Bogacz A., Cross J.H., Elger C.E., Engel J., Forsgren L., French J.A., Glynn M., Hesdorffer D.C., Lee B.I., Mathern G.W., Moshé S.L., Perucca E., Scheffer I.E., Tomson T., Watanabe M., Wiebe S. (2014). ILAE Official Report: A practical clinical definition of epilepsy.. Epilepsia.

[27] Moavero R., Santarone M.E., Galasso C., Curatolo P. (2017). Cognitive and behavioral effects of new antiepileptic drugs in pediatric epilepsy.. Brain Dev..

[28] Quon R.J., Mazanec M.T., Schmidt S.S., Andrew A.S., Roth R.M., MacKenzie T.A., Sajatovic M., Spruill T., Jobst B.C. (2020). Antiepileptic drug effects on subjective and objective cognition.. Epilepsy Behav.

[29] Gillham R.A., Williams N., Wiedmann K., Butler E., Larkin J.G., Brodie M.J. (1988). Concentration-effect relationships with carbamazepine and its epoxide on psychomotor and cognitive function in epileptic patients.. J. Neurol. Neurosurg. Psychiatry.

[30] Aldenkamp A.P., Krom M.D., Reijs R. (2003). Newer antiepileptic drugs and cognitive issues.. Epilepsia.

[31] Henske E.P., Jóźwiak S., Kingswood J.C., Sampson J.R., Thiele E.A. (2016). Tuberous sclerosis complex.. Nat. Rev. Dis. Primers.

[32] Koene L.M.C., Grondelle S.E., Proietti Onori M., Wallaard I., Kooijman N.H.R.M., Oort A., Schreiber J., Elgersma Y. (2019). Effects of antiepileptic drugs in a new TSC/mTOR‐dependent epilepsy mouse model.. Ann. Clin. Transl. Neurol..

[33] Kotulska K., Kwiatkowski D.J., Curatolo P., Weschke B., Riney K., Jansen F., Feucht M., Krsek P., Nabbout R., Jansen A.C., Wojdan K., Sijko K., Głowacka-Walas J., Borkowska J., Sadowski K., Domańska-Pakieła D., Moavero R., Hertzberg C., Hulshof H., Scholl T., Benova B., Aronica E., Ridder J., Lagae L., Jóźwiak S., Anink J., Aronica E., Benova B., Benvenuto A., Blazejczyk M., Bongaerts A., Borkowska J., Breuillard D., Chmielewski D., Curatolo P., Dabrowska M., Domańska-Pakieła D., Feucht M., Giannikou K., Głowacka-Walas J., Hamieh L., Harȩza A., Hertzberg C., Hulshof H., Huschner F., Iyer A., Jansen A., Jansen F., Janssen B., Jaworski J., Jùźwiak S., Kaczorowska-Frontczak M., Kotulska K., Krsek P., Kwiatkowski D., Lagae L., Lehmann K., Leusman A., Maćkowiak N., Mills J., Moavero R., Muelebner A., Nabbout R., Ridder J., Riney K., Sadowski K., Samueli S., Scheldeman C., Scholl T., Sciuto A., Sijko K., Słowińska M., Tempes A., Scheppingen J., Verhelle B., Vervisch J., Urbańska M., Weschke B., Wojdan K. (2021). Prevention of epilepsy in infants with tuberous sclerosis complex in the EPISTOP trial.. Ann. Neurol..

[34] Curatolo P., Verdecchia M., Bombardieri R. (2001). Vigabatrin for tuberous sclerosis complex.. Brain Dev..

[35] Curatolo P., Moavero R., van Scheppingen J., Aronica E. (2018). mTOR dysregulation and tuberous sclerosis-related epilepsy.. Expert Rev. Neurother..

[36] Curatolo P. (2015). Mechanistic target of rapamycin (mTOR) in tuberous sclerosis complex-associated epilepsy.. Pediatr. Neurol..

[37] Jaworski J., Spangler S., Seeburg D.P., Hoogenraad C.C., Sheng M. (2005). Control of dendritic arborization by the phosphoinositide-3′-kinase-Akt-mammalian target of rapamycin pathway.. J. Neurosci..

[38] von der Brelie C., Waltereit R., Zhang L., Beck H., Kirschstein T. (2006). Impaired synaptic plasticity in a rat model of tuberous sclerosis.. Eur. J. Neurosci..

[39] Bockaert J., Marin P. (2015). mTOR in brain physiology and pathologies.. Physiol. Rev..

[40] Brunklaus A., Zuberi S.M. (2014). Dravet syndrome-From epileptic encephalopathy to channelopathy.. Epilepsia.

[41] Ruffolo G., Cifelli P., Roseti C., Thom M., van Vliet E.A., Limatola C., Aronica E., Palma E. (2018). A novel GABAergic dysfunction in human Dravet syndrome.. Epilepsia.

[42] Stern W.M., Sander J.W., Rothwell J.C., Sisodiya S.M. (2017). Impaired intracortical inhibition demonstrated in vivo in people with Dravet syndrome.. Neurology.

[43] Fisher J.L. (2009). The anti-convulsant stiripentol acts directly on the GABAA receptor as a positive allosteric modulator.. Neuropharmacology.

[44] Laurie D.J., Wisden W., Seeburg P.H. (1992). The distribution of thirteen GABAA receptor subunit mRNAs in the rat brain. III. Embryonic and postnatal development.. J. Neurosci..

[45] Cherubini E., Gaiarsa J.L., Ben-Ari Y. (1991). GABA: an excitatory transmitter in early postnatal life.. Trends Neurosci..

[46] Ben-Ari Y. (2002). Excitatory actions of gaba during development: the nature of the nurture.. Nat. Rev. Neurosci..

[47] Talos D.M., Sun H., Kosaras B., Joseph A., Folkerth R.D., Poduri A., Madsen J.R., Black P.M., Jensen F.E. (2012). Altered inhibition in tuberous sclerosis and type IIb cortical dysplasia.. Ann. Neurol..

[48] Tang X., Kim J., Zhou L., Wengert E., Zhang L., Wu Z., Carromeu C., Muotri A.R., Marchetto M.C.N., Gage F.H., Chen G. (2016). KCC2 rescues functional deficits in human neurons derived from patients with Rett syndrome.. Proc. Natl. Acad. Sci..

[49] Ruffolo G., Cifelli P., Miranda-Lourenço C., De Felice E., Limatola C., Sebastião A.M., Diógenes M.J., Aronica E., Palma E. (2019). Rare diseases of neurodevelopment: Maintain the mystery or use a dazzling tool for investigation? the case of rett syndrome.. Neuroscience.

[50] Braat S., Kooy R.F. (2015). The GABAA receptor as a therapeutic target for neurodevelopmental disorders.. Neuron.

[51] Kaila K., Price T.J., Payne J.A., Puskarjov M., Voipio J. (2014). Cation-chloride cotransporters in neuronal development, plasticity and disease.. Nat. Rev. Neurosci..

[52] Ben-Ari Y., Tyzio R. (2011). Is it safe to use a diuretic to treat seizures early in development?. Epilepsy Curr..

[53] Ruffolo G., Iyer A., Cifelli P., Roseti C., Mühlebner A., van Scheppingen J., Scholl T., Hainfellner J.A., Feucht M., Krsek P., Zamecnik J., Jansen F.E., Spliet W.G.M., Limatola C., Aronica E., Palma E. (2016). Functional aspects of early brain development are preserved in tuberous sclerosis complex (TSC) epileptogenic lesions.. Neurobiol. Dis..

[54] Löscher W., Kaila K. (2022). CNS pharmacology of NKCC1 inhibitors.. Neuropharmacology.

[55] Soul J.S., Bergin A.M., Stopp C., Hayes B., Singh A., Fortuno C.R., O’Reilly D., Krishnamoorthy K., Jensen F.E., Rofeberg V., Dong M., Vinks A.A., Wypij D., Staley K.J. (2021). A pilot randomized, controlled, double‐blind trial of bumetanide to treat neonatal seizures.. Ann. Neurol..

[56] Lykke K., Töllner K., Feit P.W., Erker T., MacAulay N., Löscher W. (2016). The search for NKCC1-selective drugs for the treatment of epilepsy: Structure-function relationship of bumetanide and various bumetanide derivatives in inhibiting the human cation-chloride cotransporter NKCC1A.. Epilepsy Behav..

[57] Tang X., Drotar J., Li K., Clairmont C.D., Brumm A.S., Sullins A.J., Wu H., Liu X.S., Wang J., Gray N.S., Sur M., Jaenisch R. (2019). Pharmacological enhancement of KCC2 gene expression exerts therapeutic effects on human Rett syndrome neurons and Mecp2 mutant mice.. Sci. Transl. Med..

[58] Deidda G., Parrini M., Naskar S., Bozarth I.F., Contestabile A., Cancedda L. (2015). Reversing excitatory GABAAR signaling restores synaptic plasticity and memory in a mouse model of Down syndrome.. Nat. Med..

[59] Lucas C.J., Galettis P., Schneider J. (2018). The pharmacokinetics and the pharmacodynamics of cannabinoids.. Br. J. Clin. Pharmacol..

[60] Cifelli P., Ruffolo G., De Felice E., Alfano V., van Vliet E.A., Aronica E., Palma E. (2020). Phytocannabinoids in neurological diseases: Could they restore a physiological GABAergic transmission?. Int. J. Mol. Sci..

[61] Morano A., Cifelli P., Nencini P., Antonilli L., Fattouch J., Ruffolo G., Roseti C., Aronica E., Limatola C., Di Bonaventura C., Palma E., Giallonardo A.T. (2016). Cannabis in epilepsy: From clinical practice to basic research focusing on the possible role of cannabidivarin.. Epilepsia Open.

[62] Steel D., Symonds J.D., Zuberi S.M., Brunklaus A. (2017). Dravet syndrome and its mimics: Beyond SCN1A.. Epilepsia.

[63] Samanta D. (2021). Management of Lennox-Gastaut syndrome beyond childhood: A comprehensive review.. Epilepsy Behav.

[64] Golub V., Reddy D.S. (2021). Cannabidiol therapy for refractory epilepsy and seizure disorders.. Adv. Exp. Med. Biol..

[65] Patel A.D., Mazurkiewicz-Bełdzińska M., Chin R.F., Gil-Nagel A., Gunning B., Halford J.J., Mitchell W., Scott Perry M., Thiele E.A., Weinstock A., Dunayevich E., Checketts D., Devinsky O. (2021). Long‐term safety and efficacy of add‐on cannabidiol in patients with Lennox-Gastaut syndrome: Results of a long‐term open‐label extension trial.. Epilepsia.

[66] Devinsky O., Cross J.H., Laux L., Marsh E., Miller I., Nabbout R., Scheffer I.E., Thiele E.A., Wright S. (2017). Trial of cannabidiol for drug-resistant seizures in the dravet syndrome.. N. Engl. J. Med..

[67] Samanta D. (2022). A scoping review on cannabidiol therapy in tuberous sclerosis: Current evidence and perspectives for future development.. Epilepsy Behav..

[68] Hess E.J., Moody K.A., Geffrey A.L., Pollack S.F., Skirvin L.A., Bruno P.L., Paolini J.L., Thiele E.A. (2016). Cannabidiol as a new treatment for drug-resistant epilepsy in tuberous sclerosis complex.. Epilepsia.

[69] Aronica E., Boer K., Baybis M., Yu J., Crino P. (2007). Co-expression of cyclin D1 and phosphorylated ribosomal S6 proteins in hemimegalencephaly.. Acta Neuropathol..

[70] Bakas T., van Nieuwenhuijzen P.S., Devenish S.O., McGregor I.S., Arnold J.C., Chebib M. (2017). The direct actions of cannabidiol and 2-arachidonoyl glycerol at GABA A receptors.. Pharmacol. Res..

[71] Lattanzi S., Trinka E., Striano P., Rocchi C., Salvemini S., Silvestrini M., Brigo F. (2021). Highly purified cannabidiol for epilepsy treatment: A systematic review of epileptic conditions beyond dravet syndrome and lennox-gastaut syndrome.. CNS Drugs.

[72] Morano A., Fanella M., Albini M., Cifelli P., Palma E., Giallonardo A.T., Di Bonaventura C. (2020). Cannabinoids in the treatment of epilepsy: Current status and future prospects.. Neuropsychiatr. Dis. Treat..

[73] Klepper J., Akman C., Armeno M., Auvin S., Cervenka M., Cross H.J., De Giorgis V., Della Marina A., Engelstad K., Heussinger N., Kossoff E.H., Leen W.G., Leiendecker B., Monani U.R., Oguni H., Neal E., Pascual J.M., Pearson T.S., Pons R., Scheffer I.E., Veggiotti P., Willemsen M., Zuberi S.M., De Vivo D.C. (2020). Glut1 Deficiency Syndrome (Glut1DS): State of the art in 2020 and recommendations of the international Glut1DS study group.. Epilepsia Open.

[74] Frost G., Sleeth M.L., Sahuri-Arisoylu M., Lizarbe B., Cerdan S., Brody L., Anastasovska J., Ghourab S., Hankir M., Zhang S., Carling D., Swann J.R., Gibson G., Viardot A., Morrison D., Louise T.E., Bell J.D. (2014). The short-chain fatty acid acetate reduces appetite via a central homeostatic mechanism.. Nat. Commun..

[75] Nankova B.B., Agarwal R., MacFabe D.F., La Gamma E.F. (2014). Enteric bacterial metabolites propionic and butyric acid modulate gene expression, including CREB-dependent catecholaminergic neurotransmission, in PC12 cells--possible relevance to autism spectrum disorders.. PLoS One.

[76] Mazarati A., Sankar R. (2016). Common mechanisms underlying epileptogenesis and the comorbidities of epilepsy.. Cold Spring Harb. Perspect. Med..

[77] Ambrogini P., Torquato P., Bartolini D., Albertini M.C., Lattanzi D., Di Palma M., Marinelli R., Betti M., Minelli A., Cuppini R., Galli F. (2019). Excitotoxicity, neuroinflammation and oxidant stress as molecular bases of epileptogenesis and epilepsy-derived neurodegeneration: The role of vitamin E.. Biochim. Biophys. Acta Mol. Basis Dis..

[78] Zimmer T.S., Ciriminna G., Arena A., Anink J.J., Korotkov A., Jansen F.E., Hecke W., Spliet W.G., Rijen P.C., Baayen J.C., Idema S., Rensing N.R., Wong M., Mills J.D., Vliet E.A., Aronica E. (2020). Chronic activation of anti‐oxidant pathways and iron accumulation in epileptogenic malformations.. Neuropathol. Appl. Neurobiol..

[79] Zimmer T.S., David B., Broekaart D.W.M., Schidlowski M., Ruffolo G., Korotkov A., van der Wel N.N., van Rijen P.C., Mühlebner A., van Hecke W., Baayen J.C., Idema S., François L., van Eyll J., Dedeurwaerdere S., Kessels H.W., Surges R., Rüber T., Gorter J.A., Mills J.D., van Vliet E.A., Aronica E. (2021). Seizure-mediated iron accumulation and dysregulated iron metabolism after status epilepticus and in temporal lobe epilepsy.. Acta Neuropathol..

[80] Kim J.E., Cho K.O. (2019). Functional nutrients for epilepsy.. Nutrients.

[81] Pawlik M.J., Miziak B., Walczak A., Konarzewska A., Chrościńska-Krawczyk M., Albrecht J., Czuczwar S.J. (2021). Selected molecular targets for antiepileptogenesis.. Int. J. Mol. Sci..

[82] Ambrogini P., Minelli A., Galati C., Betti M., Lattanzi D., Ciffolilli S., Piroddi M., Galli F., Cuppini R. (2014). Post-seizure α-tocopherol treatment decreases neuroinflammation and neuronal degeneration induced by status epilepticus in rat hippocampus.. Mol. Neurobiol..

[83] Ambrogini P., Albertini M.C., Betti M., Galati C., Lattanzi D., Savelli D., Di Palma M., Saccomanno S., Bartolini D., Torquato P., Ruffolo G., Olivieri F., Galli F., Palma E., Minelli A., Cuppini R. (2018). Neurobiological correlates of alpha-tocopherol antiepileptogenic effects and microrna expression modulation in a rat model of kainate-induced seizures.. Mol. Neurobiol..

[84] Bowling F.G. (2011). Pyridoxine supply in human development.. Semin. Cell Dev. Biol..

[85] Rubinos C., Bruzzone M.J., Blodgett C., Tsai C., Patel P., Hianik R., Jadav R., Boudesseul J., Liu C., Zhu H. (2022). Association of serum pyridoxal phosphate levels with established status epilepticus.. Neurocrit. Care.

[86] Ghatge M.S., Al Mughram M., Omar A.M., Safo M.K. (2021). Inborn errors in the vitamin B6 salvage enzymes associated with neonatal epileptic encephalopathy and other pathologies.. Biochimie.

[87] Ballaz S.J., Rebec G.V. (2019). Neurobiology of vitamin C: Expanding the focus from antioxidant to endogenous neuromodulator.. Pharmacol. Res..

[88] Castaneda-Arellano R., Beas-Zarate C., Feria-Velasco A.I., Bitar-Alatorre E.W., Rivera-Cervantes M.C. (2014). From neurogenesis to neuroprotection in the epilepsy: signalling by erythropoietin.. Front. Biosci..

[89] Roseti C., Cifelli P., Ruffolo G., Barbieri E., Guescini M., Esposito V., Di Gennaro G., Limatola C., Giovannelli A., Aronica E., Palma E. (2020). Erythropoietin increases GABAA currents in human cortex from TLE patients.. Neuroscience.

[90] Jantzie L.L., Getsy P.M., Firl D.J., Wilson C.G., Miller R.H., Robinson S. (2014). Erythropoietin attenuates loss of potassium chloride co-transporters following prenatal brain injury.. Mol. Cell. Neurosci..

[91] Lin T.W., Harward S.C., Huang Y.Z., McNamara J.O. (2020). Targeting BDNF/TrkB pathways for preventing or suppressing epilepsy.. Neuropharmacology.

[92] Romoli M., Sen A., Parnetti L., Calabresi P., Costa C. (2021). Amyloid-β: A potential link between epilepsy and cognitive decline.. Nat. Rev. Neurol..

[93] Cretin B., Philippi N., Dibitonto L., Blanc F. (2017). Epilepsy at the prodromal stages of neurodegenerative diseases.. Psychol. Neuropsychiatr. Vieil..

[94] Sen A., Jette N., Husain M., Sander J.W. (2020). Epilepsy in older people.. Lancet.

[95] Amatniek J.C., Hauser W.A., DelCastillo-Castaneda C., Jacobs D.M., Marder K., Bell K., Albert M., Brandt J., Stern Y. (2006). Incidence and predictors of seizures in patients with Alzheimer’s disease.. Epilepsia.

[96] Vossel K.A., Beagle A.J., Rabinovici G.D., Shu H., Lee S.E., Naasan G., Hegde M., Cornes S.B., Henry M.L., Nelson A.B., Seeley W.W., Geschwind M.D., Gorno-Tempini M.L., Shih T., Kirsch H.E., Garcia P.A., Miller B.L., Mucke L. (2013). Seizures and epileptiform activity in the early stages of Alzheimer disease.. JAMA Neurol..

[97] Novak A., Vizjak K., Rakusa M. (2022). Cognitive impairment in people with epilepsy.. J. Clin. Med..

[98] Black L.C., Schefft B.K., Howe S.R., Szaflarski J.P., Yeh H., Privitera M.D. (2010). The effect of seizures on working memory and executive functioning performance.. Epilepsy Behav..

[99] Sen A., Capelli V., Husain M. (2018). Cognition and dementia in older patients with epilepsy.. Brain.

[100] Witt J.A., Werhahn K.J., Krämer G., Ruckes C., Trinka E., Helmstaedter C. (2014). Cognitive-behavioral screening in elderly patients with new-onset epilepsy before treatment.. Acta Neurol. Scand..

[101] Kawakami O., Koike Y., Ando T., Sugiura M., Kato H., Hiraga K., Kito H., Kondo H. (2018). Incidence of dementia in patients with adult-onset epilepsy of unknown causes.. J. Neurol. Sci..

[102] Liguori C., Costa C., Franchini F., Izzi F., Spanetta M., Cesarini E. N., Di Santo S., Manfredi N., Farotti L., Romoli M. (2019). Cognitive performances in patients affected by late-onset epilepsy with unknown etiology: A 12-month follow-up study.. Epilepsy Behav.

[103] Costa C., Parnetti L., D’Amelio M., Tozzi A., Tantucci M., Romigi A., Siliquini S., Cavallucci V., Di Filippo M., Mazzocchetti P., Liguori C., Nobili A., Eusebi P., Mercuri N.B., Calabresi P. (2016). Epilepsy, amyloid-β, and D1 dopamine receptors: A possible pathogenetic link?. Neurobiol. Aging.

[104] Costa C., Romoli M., Calabresi P. (2018). Late onset epilepsy and Alzheimer’s disease: exploring the dual pathogenic role of amyloid-β.. Brain.

[105] Sciaccaluga M., Megaro A., Bellomo G., Ruffolo G., Romoli M., Palma E., Costa C. (2021). An unbalanced synaptic transmission: Cause or consequence of the amyloid oligomers neurotoxicity?. Int. J. Mol. Sci..

[106] Yang J.W., Czech T., Felizardo M., Baumgartner C., Lubec G. (2006). Aberrant expression of cytoskeleton proteins in hippocampus from patients with mesial temporal lobe epilepsy.. Amino Acids.

[107] Choi J., Kim S.Y., Kim H., Lim B.C., Hwang H., Chae J.H., Kim K.J., Oh S., Kim E.Y., Shin J.S. (2020). Serum α-synuclein and IL-1β are increased and correlated with measures of disease severity in children with epilepsy: Potential prognostic biomarkers?. BMC Neurol..

[108] Li A., Choi Y.S., Dziema H., Cao R., Cho H.Y., Jung Y.J., Obrietan K. (2010). Proteomic profiling of the epileptic dentate gyrus.. Brain Pathol..

[109] Hussein A.M., Eldosoky M., El-Shafey M., El-Mesery M., Ali A.N., Abbas K.M., Abulseoud O.A. (2019). Effects of metformin on apoptosis and α-synuclein in a rat model of pentylenetetrazole-induced epilepsy.. Can. J. Physiol. Pharmacol..

[110] Morris M., Sanchez P.E., Verret L., Beagle A.J., Guo W., Dubal D., Ranasinghe K.G., Koyama A., Ho K., Yu G.Q., Vossel K.A., Mucke L. (2015). Network dysfunction in α ‐synuclein transgenic mice and human Lewy body dementia.. Ann. Clin. Transl. Neurol..

[111] Peters S.T., Fahrenkopf A., Choquette J.M., Vermilyea S.C., Lee M.K., Vossel K. (2020). Ablating tau reduces hyperexcitability and moderates electroencephalographic slowing in transgenic mice expressing A53T human α-synuclein.. Front. Neurol..

[112] Tweedy C., Kindred N., Curry J., Williams C., Taylor J.P., Atkinson P., Randall F., Erskine D., Morris C.M., Reeve A.K., Clowry G.J., LeBeau F.E.N. (2021). Hippocampal network hyperexcitability in young transgenic mice expressing human mutant alpha-synuclein.. Neurobiol. Dis..

[113] Lei M., Xu H., Li Z., Wang Z., O’Malley T.T., Zhang D., Walsh D.M., Xu P., Selkoe D.J., Li S. (2016). Soluble Aβ oligomers impair hippocampal LTP by disrupting glutamatergic/GABAergic balance.. Neurobiol. Dis..

[114] Nygaard H.B., Kaufman A.C., Sekine-Konno T., Huh L.L., Going H., Feldman S.J., Kostylev M.A., Strittmatter S.M. (2015). Brivaracetam, but not ethosuximide, reverses memory impairments in an Alzheimer’s disease mouse model.. Alzheimers Res. Ther..

[115] Li L., Zhang S., Zhang X., Li T., Tang Y., Liu H., Yang W., Le W. (2013). Autophagy enhancer carbamazepine alleviates memory deficits and cerebral amyloid-β pathology in a mouse model of Alzheimer’s disease.. Curr. Alzheimer Res..

[116] Mark R.J., Wesson Ashford J., Goodman Y., Mattson M.P. (1995). Anticonvulsants attenuate amyloid β-peptide neurotoxicity, Ca2+ deregulation, and cytoskeletal pathology.. Neurobiol. Aging.

[117] Ziyatdinova S., Gurevicius K., Kutchiashvili N., Bolkvadze T., Nissinen J., Tanila H., Pitkänen A. (2011). Spontaneous epileptiform discharges in a mouse model of Alzheimer’s disease are suppressed by antiepileptic drugs that block sodium channels.. Epilepsy Res..

[118] Ziyatdinova S., Viswanathan J., Hiltunen M., Tanila H., Pitkänen A. (2015). Reduction of epileptiform activity by valproic acid in a mouse model of Alzheimer’s disease is not long-lasting after treatment discontinuation.. Epilepsy Res..

[119] Shi J.Q., Wang B.R., Tian Y.Y., Xu J., Gao L., Zhao S.L., Jiang T., Xie H.G., Zhang Y.D. (2013). Antiepileptics topiramate and levetiracetam alleviate behavioral deficits and reduce neuropathology in APPswe/PS1dE9 transgenic mice.. CNS Neurosci. Ther..

[120] Williams R.S.B., Bate C. (2018). Valproic acid and its congener propylisopropylacetic acid reduced the amount of soluble amyloid-β oligomers released from 7PA2 cells.. Neuropharmacology.

[121] McQuail J.A., Beas B.S., Kelly K.B., Simpson K.L., Frazier C.J., Setlow B., Bizon J.L. (2016). NR2A-Containing NMDARs in the prefrontal cortex are required for working memory and associated with age-related cognitive decline.. J. Neurosci..

[122] Desai N.S., Rutherford L.C., Turrigiano G.G. (1999). Plasticity in the intrinsic excitability of cortical pyramidal neurons.. Nat. Neurosci..

[123] Asztely F., Kokaia M., Olofsdotter K., Örtegren U., Lindvall O. (2000). Afferent-specific modulation of short-term synaptic plasticity by neurotrophins in dentate gyrus.. Eur. J. Neurosci..

[124] Fischer A., Sananbenesi F., Schrick C., Spiess J., Radulovic J. (2002). Cyclin-dependent kinase 5 is required for associative learning.. J. Neurosci..

[125] Wu H., Lu M.H., Wang W., Zhang M.Y., Zhu Q.Q., Xia Y.Y., Xu R.X., Yang Y., Chen L.H., Ma Q.H. (2015). Lamotrigine reduces β-site aβpp-cleaving enzyme 1 protein levels through induction of autophagy.. J. Alzheimers Dis..

[126] Zhang M.Y., Zheng C.Y., Zou M.M., Zhu J.W., Zhang Y., Wang J., Liu C.F., Li Q.F., Xiao Z.C., Li S., Ma Q.H., Xu R.X. (2014). Lamotrigine attenuates deficits in synaptic plasticity and accumulation of amyloid plaques in APP/PS1 transgenic mice.. Neurobiol. Aging.

[127] Gureviciene I., Ishchenko I., Ziyatdinova S., Jin N., Lipponen A., Gurevicius K., Tanila H. (2019). Characterization of epileptic spiking associated with brain amyloidosis in APP/PS1 mice.. Front. Neurol..

[128] Sanchez P.E., Zhu L., Verret L., Vossel K.A., Orr A.G., Cirrito J.R., Devidze N., Ho K., Yu G.Q., Palop J.J., Mucke L. (2012). Levetiracetam suppresses neuronal network dysfunction and reverses synaptic and cognitive deficits in an Alzheimer’s disease model.. Proc. Natl. Acad. Sci..

[129] Lynch B.A., Lambeng N., Nocka K., Kensel-Hammes P., Bajjalieh S.M., Matagne A., Fuks B. (2004). The synaptic vesicle protein SV2A is the binding site for the antiepileptic drug levetiracetam.. Proc. Natl. Acad. Sci..

[130] Kaminski R.M., Matagne A., Leclercq K., Gillard M., Michel P., Kenda B., Talaga P., Klitgaard H. (2008). SV2A protein is a broad-spectrum anticonvulsant target: Functional correlation between protein binding and seizure protection in models of both partial and generalized epilepsy.. Neuropharmacology.

[131] Nagarkatti N., Deshpande L.S., DeLorenzo R.J. (2008). Levetiracetam Inhibits both ryanodine and IP3 receptor activated calcium induced calcium release in hippocampal neurons in culture.. Neurosci. Lett..

[132] Angehagen M., Margineanu D.G., Ben-Menachem E., Rönnbäck L., Hansson E., Klitgaard H. (2003). Levetiracetam reduces caffeine-induced Ca2+transients and epileptiform potentials in hippocampal neurons.. Neuroreport.

[133] Demuro A., Parker I., Stutzmann G.E. (2010). Calcium signaling and amyloid toxicity in Alzheimer disease.. J. Biol. Chem..

[134] Bezprozvanny I., Mattson M.P. (2008). Neuronal calcium mishandling and the pathogenesis of Alzheimer’s disease.. Trends Neurosci..

[135] Leal S.L., Landau S.M., Bell R.K., Jagust W.J. (2017). Hippocampal activation is associated with longitudinal amyloid accumulation and cognitive decline.. eLife.

[136] Sendrowski K., Sobaniec W., Stasiak-Barmuta A., Sobaniec P., Popko J. (2015). Study of the protective effects of nootropic agents against neuronal damage induced by amyloid-beta (fragment 25-35) in cultured hippocampal neurons.. Pharmacol. Rep..

[137] Sanz-Blasco S., Piña-Crespo J.C., Zhang X., McKercher S.R., Lipton S.A. (2016). Levetiracetam inhibits oligomeric Aβ-induced glutamate release from human astrocytes.. Neuroreport.

[138] Sen A., Akinola M., Tai X.Y., Symmonds M., Davis Jones G., Mura S., Galloway J., Hallam A., Chan J.Y.C., Koychev I., Butler C., Geddes J., Van Der Putt R., Thompson S., Manohar S.G., Frangou E., Love S., McShane R., Husain M. (2021). An Investigation of Levetiracetam in Alzheimer’s Disease (ILiAD): A double-blind, placebo-controlled, randomised crossover proof of concept study.. Trials.

[139] Vezzani A., Aronica E., Mazarati A., Pittman Q.J. (2013). Epilepsy and brain inflammation.. Exp. Neurol..

[140] Upaganlawar A.B., Wankhede N.L., Kale M.B., Umare M.D., Sehgal A., Singh S., Bhatia S., Al-Harrasi A., Najda A., Nurzyńska-Wierdak R., Bungau S., Behl T. (2021). Interweaving epilepsy and neurodegeneration: Vitamin E as a treatment approach.. Biomed. Pharmacother..

[141] Di Filippo M., Picconi B., Costa C., Bagetta V., Tantucci M., Parnetti L., Calabresi P. (2006). Pathways of neurodegeneration and experimental models of basal ganglia disorders: Downstream effects of mitochondrial inhibition.. Eur. J. Pharmacol..

[142] Mattson M.P., Gleichmann M., Cheng A. (2008). Mitochondria in neuroplasticity and neurological disorders.. Neuron.

[143] Dong X., Wang Y., Qin Z. (2009). Molecular mechanisms of excitotoxicity and their relevance to pathogenesis of neurodegenerative diseases.. Acta Pharmacol. Sin..

[144] Papa S., De Rasmo D. (2013). Complex I deficiencies in neurological disorders.. Trends Mol. Med..

[145] Costa C., Belcastro V., Tozzi A., Di Filippo M., Tantucci M., Siliquini S., Autuori A., Picconi B., Spillantini M.G., Fedele E., Pittaluga A., Raiteri M., Calabresi P. (2008). Electrophysiology and pharmacology of striatal neuronal dysfunction induced by mitochondrial complex I inhibition.. J. Neurosci..

[146] Costa C., Tozzi A., Luchetti E., Siliquini S., Belcastro V., Tantucci M., Picconi B., Ientile R., Calabresi P., Pisani F. (2010). Electrophysiological actions of zonisamide on striatal neurons: Selective neuroprotection against complex I mitochondrial dysfunction.. Exp. Neurol..

[147] Biton V. (2007). Clinical pharmacology and mechanism of action of zonisamide.. Clin. Neuropharmacol..

[148] Iuvone T., Esposito G., Esposito R., Santamaria R., Di Rosa M., Izzo A.A. (2004). Neuroprotective effect of cannabidiol, a non-psychoactive component from Cannabis sativa, on beta-amyloid-induced toxicity in PC12 cells.. J. Neurochem..

[149] Esposito G., De Filippis D., Carnuccio R., Izzo A.A., Iuvone T. (2006). The marijuana component cannabidiol inhibits β-amyloid-induced tau protein hyperphosphorylation through Wnt/β-catenin pathway rescue in PC12 cells.. J. Mol. Med..

[150] Esposito G., Scuderi C., Valenza M., Togna G.I., Latina V., De Filippis D., Cipriano M., Carratù M.R., Iuvone T., Steardo L. (2011). Cannabidiol reduces Aβ-induced neuroinflammation and promotes hippocampal neurogenesis through PPARγ involvement.. PLoS One.

[151] Wolf S.A., Bick-Sander A., Fabel K., Leal-Galicia P., Tauber S., Ramirez-Rodriguez G., Müller A., Melnik A., Waltinger T.P., Ullrich O., Kempermann G. (2010). Cannabinoid receptor CB1 mediates baseline and activity-induced survival of new neurons in adult hippocampal neurogenesis.. Cell Commun. Signal..

[152] Scuderi C., Steardo L., Esposito G. (2014). Cannabidiol promotes amyloid precursor protein ubiquitination and reduction of beta amyloid expression in SHSY5YAPP+ cells through PPARγ involvement.. Phytother. Res..

[153] Martín-Moreno A.M., Reigada D., Ramírez B.G., Mechoulam R., Innamorato N., Cuadrado A., de Ceballos M.L. (2011). Cannabidiol and other cannabinoids reduce microglial activation in vitro and in vivo: relevance to Alzheimer’s disease.. Mol. Pharmacol..

[154] Aso E., Sánchez-Pla A., Vegas-Lozano E., Maldonado R., Ferrer I. (2014). Cannabis-based medicine reduces multiple pathological processes in AβPP/PS1 mice.. J. Alzheimers Dis..

[155] Watt G., Shang K., Zieba J., Olaya J., Li H., Garner B., Karl T. (2020). Chronic treatment with 50 mg/kg cannabidiol improves cognition and moderately reduces aβ40 levels in 12-month-old male AβPPswe/PS1ΔE9 transgenic mice.. J. Alzheimers Dis..

[156] Schubert D., Kepchia D., Liang Z., Dargusch R., Goldberg J., Maher P. (2019). Efficacy of cannabinoids in a pre-clinical drug-screening platform for Alzheimer’s Disease.. Mol. Neurobiol..

[157] Dash R., Ali M.C., Jahan I., Munni Y.A., Mitra S., Hannan M.A., Timalsina B., Oktaviani D.F., Choi H.J., Moon I.S. (2021). Emerging potential of cannabidiol in reversing proteinopathies.. Ageing Res. Rev..

[158] Libro R., Diomede F., Scionti D., Piattelli A., Grassi G., Pollastro F., Bramanti P., Mazzon E., Trubiani O. (2016). Cannabidiol modulates the expression of alzheimer’s disease-related genes in mesenchymal stem cells.. Int. J. Mol. Sci..

[159] Vallée A., Lecarpentier Y., Guillevin R., Vallée J.N. (2017). Effects of cannabidiol interactions with Wnt/β-catenin pathway and PPARγ on oxidative stress and neuroinflammation in Alzheimer’s disease.. Acta Biochim. Biophys. Sin..

[160] Sutula T.P., Hagen J., Pitkänen A. (2003). Do epileptic seizures damage the brain?. Curr. Opin. Neurol..

[161] Walker M.C., White H.S., Sander J.W.S. (2002). Disease modification in partial epilepsy.. Brain.

[162] Milward A.J., Meldrum B.S., Mellanby J.H. (1999). Forebrain ischaemia with CA1 cell loss impairs epileptogenesis in the tetanus toxin limbic seizure model.. Brain.

[163] Sweatt J.D. (2004). Mitogen-activated protein kinases in synaptic plasticity and memory.. Curr. Opin. Neurobiol..

[164] Calabresi P., Centonze D., Pisani A., Cupini L.M., Bernardi G. (2003). Synaptic plasticity in the ischaemic brain.. Lancet Neurol..

[165] Calabresi P., Cupini L.M., Centonze D., Pisani F., Bernardi G. (2003). Antiepileptic drugs as a possible neuroprotective strategy in brain ischemia.. Ann. Neurol..

[166] Costa C., Leone G., Saulle E., Pisani F., Bernardi G., Calabresi P. (2004). Coactivation of GABA(A) and GABA(B) receptor results in neuroprotection during in vitro ischemia.. Stroke.

[167] Calabresi P., Marti M., Picconi B., Saulle E., Costa C., Centonze D., Pisani F., Bernardi G. (2003). Lamotrigine and remacemide protect striatal neurons against in vitro ischemia: an electrophysiological study.. Exp. Neurol..

[168] Costa C., Martella G., Picconi B., Prosperetti C., Pisani A., Di Filippo M., Pisani F., Bernardi G., Calabresi P. (2006). Multiple mechanisms underlying the neuroprotective effects of antiepileptic drugs against in vitro ischemia.. Stroke.

[169] Lindberger M., Tomson T., Ståhle L. (2002). Microdialysis sampling of carbamazepine, phenytoin and phenobarbital in subcutaneous extracellular fluid and subdural cerebrospinal fluid in humans: an in vitro and in vivo study of adsorption to the sampling device.. Pharmacol. Toxicol..

[170] Christensen J., Højskov C.S., Dam M., Poulsen J.H. (2001). Plasma concentration of topiramate correlates with cerebrospinal fluid concentration.. Ther. Drug Monit..

[171] Davis R., Peters D.H., McTavish D. (1994). Valproic Acid.. Drugs.

[172] Ren M., Leng Y., Jeong M., Leeds P.R., Chuang D.M. (2004). Valproic acid reduces brain damage induced by transient focal cerebral ischemia in rats: potential roles of histone deacetylase inhibition and heat shock protein induction.. J. Neurochem..

[173] Eyal S., Yagen B., Sobol E., Altschuler Y., Shmuel M., Bialer M. (2004). The activity of antiepileptic drugs as histone deacetylase inhibitors.. Epilepsia.

[174] Xuan A., Long D., Li J., Ji W., Hong L., Zhang M., Zhang W. (2012). Neuroprotective effects of valproic acid following transient global ischemia in rats.. Life Sci..

[175] Zhu S., Zhang Z., Jia L., Zhan K., Wang L., Song N., Liu Y., Cheng Y., Yang Y., Guan L., Min D., Yang G. (2019). Valproic acid attenuates global cerebral ischemia/reperfusion injury in gerbils via anti-pyroptosis pathways.. Neurochem. Int..

[176] Naseh M., Bayat M., Akbari S., Vatanparast J., Shabani M., Haghighi A.B., Haghani M. (2022). Neuroprotective effects of sodium valproate on hippocampal cell and volume, and cognitive function in a rat model of focal cerebral ischemia.. Physiol. Behav..

[177] Helmstaedter C., Witt J.A. (2013). The longer-term cognitive effects of adjunctive antiepileptic treatment with lacosamide in comparison with lamotrigine and topiramate in a naturalistic outpatient setting.. Epilepsy Behav..

[178] Witt J.A., Elger C.E., Helmstaedter C. (2015). Adverse cognitive effects of antiepileptic pharmacotherapy: Each additional drug matters.. Eur. Neuropsychopharmacol..

[179] Ikonomidou C., Turski L. (2010). Antiepileptic drugs and brain development.. Epilepsy Res..

[180] Sgobio C., Ghiglieri V., Costa C., Bagetta V., Siliquini S., Barone I., Di Filippo M., Gardoni F., Gundelfinger E.D., Di Luca M., Picconi B., Calabresi P. (2010). Hippocampal synaptic plasticity, memory, and epilepsy: effects of long-term valproic acid treatment.. Biol. Psychiatry.

[181] Mazzocchetti P., Tantucci M., Bastioli G., Calabrese V., Di Filippo M., Tozzi A., Calabresi P., Costa C. (2018). Lacosamide protects striatal and hippocampal neurons from in vitro ischemia without altering physiological synaptic plasticity.. Neuropharmacology.

[182] Mazzocchetti P., Mancini A., Sciaccaluga M., Megaro A., Bellingacci L., Di Filippo M., Cesarini E.N., Romoli M., Carrano N., Gardoni F., Tozzi A., Calabresi P., Costa C. (2020). Low doses of Perampanel protect striatal and hippocampal neurons against in vitro ischemia by reversing the ischemia-induced alteration of AMPA receptor subunit composition.. Neurobiol. Dis..

[183] Rogawski M.A., Tofighy A., White H.S., Matagne A., Wolff C. (2015). Current understanding of the mechanism of action of the antiepileptic drug lacosamide.. Epilepsy Res..

[184] Nakajima M., Suda S., Sowa K., Sakamoto Y., Nito C., Nishiyama Y., Aoki J., Ueda M., Yokobori S., Yamada M., Yokota H., Okada T., Kimura K. (2018). AMPA receptor antagonist perampanel ameliorates post-stroke functional and cognitive impairments.. Neuroscience.

[185] Niu H.X., Wang J.Z., Wang D.L., Miao J.J., Li H., Liu Z.G., Yuan X., Liu W., Zhou J.R. (2018). The orally active noncompetitive AMPAR antagonist perampanel attenuates focal cerebral ischemia injury in rats.. Cell. Mol. Neurobiol..

[186] Maggio N., Lenz M., Vlachos A. (2015). Ischemic long-term-potentiation (iLTP): perspectives to set the threshold of neural plasticity toward therapy.. Neural Regen. Res..

[187] Takahiro Hayakawa (1994). Yoshihisa Higuchi; Hiroyuki Nigami; Haruo Hattori, Zonisamide reduces hypoxic-ischemic brain damage in neonatal rats irrespective of its anticonvulsive effect.. Eur. J. Pharmacol..

[188] Minato H., Kikuta C., Fujitani B., Masuda Y. (1997). Protective effect of zonisamide, an antiepileptic drug, against transient focal cerebral ischemia with middle cerebral artery occlusion-reperfusion in rats.. Epilepsia.

[189] Owen A.J., Ijaz S., Miyashita H., Wishart T., Howlett W., Shuaib A. (1997). Zonisamide as a neuroprotective agent in an adult gerbil model of global forebrain ischemia: a histological, in vivo microdialysis and behavioral study.. Brain Res..

[190] Costa C., Tozzi A., Siliquini S., Galletti F., Cardaioli G., Tantucci M., Pisani F., Calabresi P. (2011). A critical role of NO/cGMP/] PKG dependent pathway in hippocampal post-ischemic LTP: Modulation by zonisamide.. Neurobiol. Dis..

[191] Hayakawa K., Mishima K., Fujiwara M. (2010). Therapeutic potential of non-psychotropic cannabidiol in ischemic stroke.. Pharmaceuticals.

[192] Mishima K., Hayakawa K., Abe K., Ikeda T., Egashira N., Iwasaki K., Fujiwara M. (2005). Cannabidiol prevents cerebral infarction via a serotonergic 5-hydroxytryptamine1A receptor-dependent mechanism.. Stroke.

[193] Alvarez F.J., Lafuente H., Carmen R.M., Mielgo V.E., Gastiasoro E., Rueda M., Pertwee R.G., Castillo A.I., Romero J., Martínez-Orgado J. (2008). Neuroprotective effects of the nonpsychoactive cannabinoid cannabidiol in hypoxic-ischemic newborn piglets.. Pediatr. Res..

[194] Castillo A., Tolón M.R., Fernández-Ruiz J., Romero J., Martinez-Orgado J. (2010). The neuroprotective effect of cannabidiol in an in vitro model of newborn hypoxic-ischemic brain damage in mice is mediated by CB2 and adenosine receptors.. Neurobiol. Dis..

[195] Lafuente H., Alvarez F.J., Pazos M.R., Alvarez A., Rey-Santano M.C., Mielgo V., Murgia-Esteve X., Hilario E., Martinez-Orgado J. (2011). Cannabidiol reduces brain damage and improves functional recovery after acute hypoxia-ischemia in newborn pigs.. Pediatr. Res..

[196] Pazos M.R., Cinquina V., Gómez A., Layunta R., Santos M., Fernández-Ruiz J., Martínez-Orgado J. (2012). Cannabidiol administration after hypoxia-ischemia to newborn rats reduces long-term brain injury and restores neurobehavioral function.. Neuropharmacology.

[197] Pazos M.R., Mohammed N., Lafuente H., Santos M., Martínez-Pinilla E., Moreno E., Valdizan E., Romero J., Pazos A., Franco R., Hillard C.J., Alvarez F.J., Martínez-Orgado J. (2013). Mechanisms of cannabidiol neuroprotection in hypoxic-ischemic newborn pigs: Role of 5HT1A and CB2 receptors.. Neuropharmacology.

[198] Mechoulam R., Peters M., Murillo-Rodriguez E., Hanuš L.O. (2007). Cannabidiol-recent advances.. Chem. Biodivers..

[199] Ceprián M., Jiménez-Sánchez L., Vargas C., Barata L., Hind W., Martínez-Orgado J. (2017). Cannabidiol reduces brain damage and improves functional recovery in a neonatal rat model of arterial ischemic stroke.. Neuropharmacology.

[200] Meyer E., Bonato J.M., Mori M.A., Mattos B.A., Guimarães F.S., Milani H., de Campos A.C., de Oliveira R.M.W. (2021). Cannabidiol confers neuroprotection in rats in a model of transient global cerebral ischemia: Impact of hippocampal synaptic neuroplasticity.. Mol. Neurobiol..

[201] Bakker A., Krauss G.L., Albert M.S., Speck C.L., Jones L.R., Stark C.E., Yassa M.A., Bassett S.S., Shelton A.L., Gallagher M. (2012). Reduction of hippocampal hyperactivity improves cognition in amnestic mild cognitive impairment.. Neuron.

[202] Dirani M., Nasreddine W., Abdulla F., Beydoun A. (2014). Seizure control and improvement of neurological dysfunction in Lafora disease with perampanel.. Epilepsy Behav. Case Rep..

[203] IJff D.M., van Veenendaal T.M., Majoie H.J.M., de Louw A.J.A., Jansen J.F.A., Aldenkamp A.P. (2015). Cognitive effects of lacosamide as adjunctive therapy in refractory epilepsy.. Acta Neurol. Scand..

